# Traditional Taxonomic Groupings Mask Evolutionary History: A Molecular Phylogeny and New Classification of the Chromodorid Nudibranchs

**DOI:** 10.1371/journal.pone.0033479

**Published:** 2012-04-10

**Authors:** Rebecca Fay Johnson, Terrence M. Gosliner

**Affiliations:** Department of Invertebrate Zoology and Geology, California Academy of Sciences, San Francisco, California, United States of America,; J. Craig Venter Institute, United States of America

## Abstract

Chromodorid nudibranchs (16 genera, 300+ species) are beautiful, brightly colored sea slugs found primarily in tropical coral reef habitats and subtropical coastal waters. The chromodorids are the most speciose family of opisthobranchs and one of the most diverse heterobranch clades. Chromodorids have the potential to be a model group with which to study diversification, color pattern evolution, are important source organisms in natural products chemistry and represent a stunning and widely compelling example of marine biodiversity. Here, we present the most complete molecular phylogeny of the chromodorid nudibranchs to date, with a broad sample of 244 specimens (142 new), representing 157 (106 new) chromodorid species, four actinocylcid species and four additional dorid species utilizing two mitochondrial markers (16s and COI). We confirmed the monophyly of the Chromodorididae and its sister group relationship with the Actinocyclidae. We were also able to, for the first time, test generic monophyly by including more than one member of all 14 of the non-monotypic chromodorid genera. Every one of these 14 traditional chromodorid genera are either non-monophyletic, or render another genus paraphyletic. Additionally, both the monotypic genera *Verconia* and *Diversidoris* are nested within clades. Based on data shown here, there are three individual species and five clades limited to the eastern Pacific and Atlantic Oceans (or just one of these ocean regions), while the majority of chromodorid clades and species are strictly Indo-Pacific in distribution. We present a new classification of the chromodorid nudibranchs. We use molecular data to untangle evolutionary relationships and retain a historical connection to traditional systematics by using generic names attached to type species as clade names.

## Introduction

The chromodorid nudibranchs are a brightly colored, morphologically diverse and species-rich group of sea slugs. Edmunds' [1] stated that, “Chromodorid nudibranchs are among the most gorgeously colored of all animals”. The over 300 described species are primarily found in tropical and subtropical waters, as members of coral reef communities, specifically associated with their sponge prey. The chromodorids are the most speciose family of opisthobranchs; their numbers rival the most diverse gastropod clades, e.g Cypraeidae (∼200 spp.), Conidae (∼500 spp.), Muricidae (∼1600 spp.) and Turridae (∼4,000 spp.). The beauty and diversity of the chromodorid nudibranchs has attracted attention from scientists, divers and underwater photographers. Despite this growing interest and subsequent increased exploration into their ecology [2]–[5], natural products chemistry [6]–[10], color pattern evolution [11]–[15] and natural history [16]–[18], there is not a comprehensive, well-supported phylogeny of the chromodorid nudibranchs. Species misidentifications in ecological and chemical studies can lead to incorrect conclusions, especially when one species name represents more than one lineage [10], [19]–[22]. In phylogenetic studies, genera are often represented by a limited number of species, in many cases the only the type species [23], but little attention has been paid to the risk of drawing incorrect conclusions if the generic groupings that serve as proxies of relationship are not monophyletic [24], [25]. More comprehensive understanding of ecological, biogoegraphical and evolutionary patterns in the chromodorids is hindered by the lack of a detailed molecular phylogeny of this group and continued use of known non-monotypic names to convey relationship and information.

**Figure 1:**
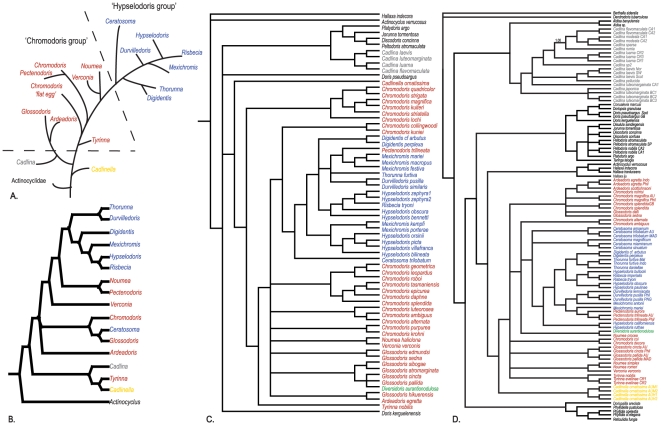
Previous Phylogenetic Hypotheses. A. ‘Phylogenetic scenario’ for the chromodorid genera modified from [5], [26]. B. Morphological phylogeny of generic representatives for the Chromodorididae [13]. C. Combined 16s and COI phylogram of the Chromodorididae from [27]. D. Combined 16s and COI phylogram of the Chromodorididae and Cadlinidae from [28]. Rudman's ‘*Chromodoris* group’ in red, ‘*Hypselodoris* group’ in blue, *Cadlinella* in yellow, *Diversidoris* (not included in [5], [13], [26]), *Cadlina* in grey and other dorids in black.

**Figure 2:**
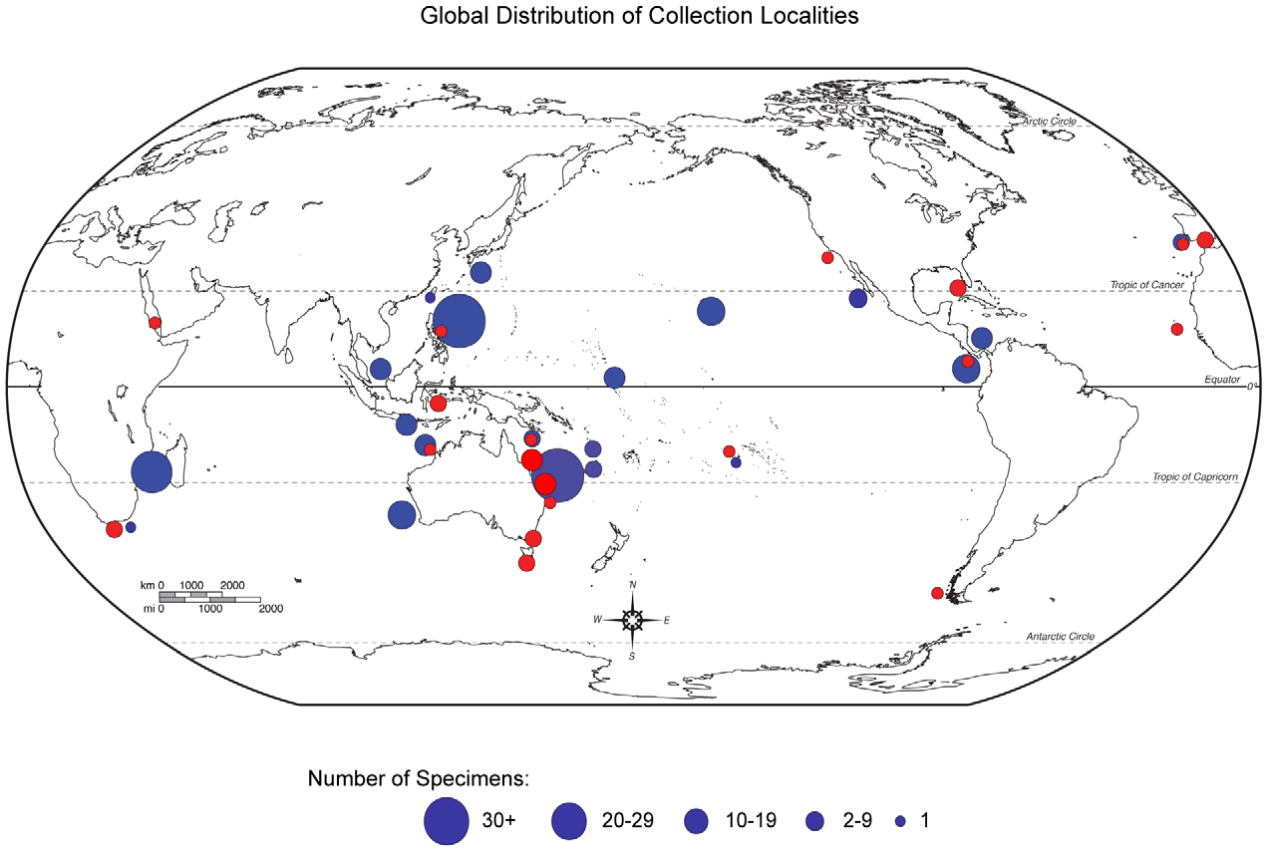
Map of collection localities and numbers of specimens. *New collections (from this contribution and* [*28*] *in blue. GenBank specimens in red. Size of circle represents number of specimens collected in each region. Specimen details in Supplementary Table S1).*

Historically, the classification of the Chromodorididae has been based on morphological similarity, primarily radular morphology, and has included species and genera thought to be closely related to *Chromodoris*, *Hypselodoris* and *Cadlina* [26]. But there has been substantial debate over the inclusion of *Cadlina* in the Chromodorididae. The majority of the molecular evidence and re-evaluated morphological data suggest *Cadlina* should not be a considered a member of the Chromodorididae, but instead the Cadlinidae, and it will not be included here [27], [28]. Most previous phylogenetic studies that have focused solely on chromodorids have used only morphological data to understand species level relationships [13], [26], [29]–[31]. Exceptions include [17], [27], [28], who used molecular data. Additionally, most phylogenetic hypotheses of relationships in the chromodorid nudibranchs either focused on only one genus, or used genera, or one representative of a genus as terminal taxa. Rudman [26] and Rudman and Berquist [5] used composite representatives for each genus when building their phylogeny. Gosliner & Johnson [13] used published data on the type species of each chromodorid genus in their preliminary phylogeny of the family. They did not include any other species to test the monophyly of any of the genera. Valdés [23], in a morphological phylogeny of all dorid nudibranchs found the chromodorids, represented by ‘*Cadlina*’ and ‘*Chromodoris*’ to be monophyletic and sister to the actinocyclids. Turner and Wilson [27] presented the first molecular phylogeny of the chromodorid nudibranchs. Their study included fifty-six chromodorid species, the majority of which were from the eastern Atlantic (EA) and eastern and southern Australia, and for the first time, at least one species of each of the chromodorid genera. See [17], [27], [28 32]–[37] for all details on all specimen data used. They were the first to explicitly test the monophyly of most chromodorid genera. Although, they were the first to find evidence for non-monophyly in *Chromodoris*, *Hypselodoris* and *Mexichromis* [27], they found little support for major clades and did not propose any changes to current classifications. Johnson [28] confirmed these findings and with the addition of sequences from fifty-five additional chromodorid and cadlinid specimens (41 species, 26 species of which had not been included in previous molecular studies) and more dorid taxa, also showed *Glossodoris* and *Noumea* to be polyphyletic [28] (for a review of previous hypotheses see Figure 1). We expand on this preliminary research and include more than one species from each genus (except monotypic genera), and include for the first time, the type species of every chromodorid genus. The Indo-Pacific (IP) is home to the greatest diversity of chromodorid nudibranchs [38]–[40] and yet the majority of taxa from this region has not been included in any molecular studies of the group, until now (Figure 2). One of the main objectives of this work is to advance chromodorid systematics and to provide a phylogenetic framework with which our traditional use of morphological data can be examined.

The goals of this contribution are:(1) generate a phylogeny that tests the species level relationships of the chromodorid nudibranchs and confirms the monophyly of the Chromodorididae, (2) assess the phylogenetic validity of the chromodorid genera, and (3) propose a new classification for the chromodorid nudibranchs that reflects their relationships.

## Materials and Methods

### Selection of Taxa

In this study and a companion study [28], thanks to targeted collecting trips, dedicated collectors and DNA extracted from museum collections, we were able to include specimens from throughout the Indo-Pacific (IP), the eastern Pacific (EP) and West Atlantic (WA) (Figure 2 and Table S1). We use the term Indo-Pacific to define the biogeographic region including the tropical and subtropical regions of the Indian Ocean (from the Red Sea to the east coast of South Africa) and both the western and central Pacific, but not the tropical eastern Pacific [40]. Museum collections are an invaluable resource for biodiversity studies [41]. We have found existing natural history collections can reduce the need for additional collecting. Our study, combined with data from [28] and GenBank, is unique in its wide taxonomic and geographic sampling. Because we have included both the type species of every genus and additional species of all 14 of the non-monotypic genera, we can test the monophyly every genus in the family (Table S1).

We directly sequenced 142 specimens representing 106 species. We combined these new data with all available sequences on GenBank (Table S1). Specimens and data from Johnson [28], GenBank accession numbers beginning with EU, are included with new data for Figure 2, but are not treated as new in the numbers of specimens sequenced for this study. In total, we analyzed data from 244 chromodorid specimens, four actinocyclid species and four additional dorid nudibranch species for a total of 165 species and 252 individual specimens. We used *Doris kerguelensis* as the outgroup based on preliminary analyses [28]. The chromodorid species include at least one species from all of the genera currently classified in the family Chromodorididae. The number of species included in this analysis compared to the number of described species per genus is as follows: *Ardeadoris* (2/2), *Cadlinella* (2/3), *Ceratosoma* (9/13, two undescribed), *Chromodoris* (50/88, two undescribed), *Digidentis* (3/4), *Diversidoris (1/1)*, *Durvilledoris* (3/4), *Glossodoris* (17/30, two undescribed), *Hypselodoris* (30/59, two undescribed), *Mexichromis* (7/12), *Noumea* (12/22), *Pectenodoris* (2/2), *Risbecia* (3/5), *Thorunna* (8/12), *Tyrinna* (2/2) and *Verconia* (1/1) (S1). All sequences taken from GenBank are listed with GB following the species name. We also included COI sequence from two specimens from the Moorea BioCode project in our analyses (http://bscit.berkeley.edu/biocode/). We have examined all of the new specimens included here and they are deposited in natural history museums, as indicated by catalog numbers. We never combined sequences from different individuals into chimeras representing one species; specimens included in these analyses are treated as individuals.

### Ethics Statement

The majority of the specimens used in this study are part of the California Academy of Sciences Invertebrate Zoology (CASIZ) collection. We had the permission of CASIZ to take tissue samples from specimens for DNA analysis. As stated in the CASIZ collections policy: ‘No specimens will be accessioned without adequate labeling, collection notes, field notes, or other locality information, nor without appropriate legal documentation (collecting permits, export permits from country of origin, etc.) when applicable.’ We also included DNA extracted for five specimens currently deposited in the Museum National d'Histoire Naturelle (Paris Museum) and the Western Australian Museum. These tissues samples were collected during joint field trips under the agreement that the tissue could be sequenced at the California Academy of Sciences, while the specimens would remain at the respective museum. All other data used is from GenBank or the Moorea BioCode Database.

### Preservation, Extraction and Amplification

Most of our samples were collected especially for molecular work and were preserved accordingly, either in 95% ETOH, SED buffer (saturated NaCl solution with EDTA and DMSO) or frozen. In addition to the specimens collected specifically for molecular study, we were also able to use museum material that was, either preserved in 70–75% EtOH or the original fixation method is unknown.

### DNA extraction and PCR amplification

We initially used standard phenol-cholorform extractions [42], [43] to extract genomic DNA and also used the Dneasy spin column extraction method (Qiagen) to extract genomic DNA from the majority of our samples. We used universal primers to amplify, using PCR, double-stranded products from both the cytochrome oxidase 1 (COI) and 16S mitochondrial genes. We targeted a 658 bp fragment of COI using Folmer et al's [44] universal primers and for 16s sequences, We used Palumbi's [45] 16Sar and 16Sbr primers. We carried out the polymerase chain reaction in 25 µL reactions with one µL of genomic DNA template. We used the second (200 µL) elution from my extractions in Dneasy AE buffer as the DNA template in most reactions. If the amplification was difficult we used one µL of the first elution. For the phenol-cloroform and chelex extractions, we used dilutions of 1∶25 or 1∶50. No matter the extraction method used, we included 2.5 µL of 10× PCR buffer, 0.5 µL dNTPs (10 mM stock), 0.25 µL of each primer (25 uM stock), 0.75–0.85 µL MgCl (50 mM stock), 0.25 µL Taq (5 units/µL )-Apex, Biolase,USB HotStart- and 19.5 µL of ddH2O in each reaction tube. We ran all of the reactions on a BioRad MyCycler™ Thermocycler (software version 1.065, Bio-Rad Laboratories). COI segments were amplified with the following parameters: an initial denaturation at 94°C for three minutes, then, 39 cycles of denaturation at 94°C for 30 seconds; annealing at 46°C for 30 seconds; extension at 72°C for 60 seconds, these cycles were followed by extension at 72°C for five minutes. Partial 16s sequences were amplified with the following parameters: an initial denaturation at 94°C for three minutes, then 39 cycles of denaturation at 94°C for 30 seconds; annealing at 50–52°C for 30 seconds; extension at 72°C for 60 seconds, these cycles were followed by extension at 72°C for five minutes and 25°C for 60 seconds. We used electrophoresis to view PCR products on 0.8% TBE or TAE agarose gel stained with ethidium bromide. We cleaned successful PCR products with ExoSap-It (USB Scientific) following each product's standard protocol.

### Sequencing

The cleaned, PCR products were copied and labeled with fluorescently dye-terminators (Big Dye 3.1 ABI) in 10 µL reactions. Each reaction contained 0.5–2 µL of cleaned PCR product, 1.63 µL of 5× reaction buffer, .5 µL of primer (10 mM stock), 0.5 µL–0.75 µL of Big Dye and water to 10 µL . These reactions were run on a Perkin Elmer 9600-GeneAmp PCR System or a BioRad MyCycler™ Thermocycler (software version 1.065, Bio-Rad Laboratories). The resulting labeled, single stranded DNA was precipitated by addition of 2.5 µL of EDTA and sequential washing and pelleting in (centrifuge details) with 100% and then 70% EtOH. The pelleted DNA was denautured for two minutes at 94°C in 13–15 µL of HiDi formamide (Applied BioSystems). The denatured, labeled DNA fragments were sequenced in both directions on the ABI 3100 and 3130 Genetic Analyzer in the Center for Comparative Genomics (formerly the Osher Laboratory for Molecular Systematics) at the California Academy of Sciences.

### Sequence editing and alignment

We assembled, edited and removed primer strands from forward and reverse strands for each gene fragment sequenced using Sequencher (ver. 4.7. GeneCodes Corporation) and Genious 3.0-5.3.3 (Biomatters). We aligned the COI sequences by eye and translated the base pair data into amino acids to using MacClade 4.08 [46] to confirm alignment accuracy. We aligned 16s sequences with MUSCLE [47]. We then further optimized the alignments by eye using both MacClade [46] and Genious 3.0-5.3.3 (Biomatters).

### Saturation

We tested for saturation or multiple substitutions at the same site by plotting the absolute number of transitions and transversions at each codon position (1^st^, 2^nd^, 3^rd^) for COI and at each base pair for 16s against both uncorrected p distance and log det using PAUP [48] and Excel (Plots not shown).

### Data sets and Phylogenetic Reconstruction

Sequence data for both genes was not obtained for every specimen we studied. We worked with two main data sets, because we wanted to test the effect of missing data on the resulting phylogeny: The two data sets were: 1) Combined 16s and COI for specimens with sequence data for both genes, 2) All 16s and COI data for all specimens (Table S1.) Both of these data sets were analyzed both including and excluding variable characters in the 16s alignment. For all of these analyses we used *Doris kerguelensis* as the outgroup.

We determined the best-fit model of evolution for each codon position for COI (1^st^, 2^nd^ 3^rd^) and the 16s fragment using the AIC selection from Mr. ModelTest ver.2 [49] for each dataset. We ran Bayesian phylogenetic analyses using Mr. Bayes 3.1.2 [50]–[52]. We ran a Monte Carlo-Metropolis simulation for 50,000,000 generations for each dataset, with trees sampled every 1000 generations. Data was partitioned by gene and by codon position in all combined analyses. We ran one analysis of two runs of six chains for all data sets. All other settings remained in the default and all parameters were unlinked to allow each partition to vary independently (Mr. Bayes 3.12 manual, http://mrbayes.csit.fsu.edu/manual.php) All trees saved before convergence of the runs and stationarity of likelihood values were discarded. We determined convergence and stationarity by plotting tree number against likelihood scores for each run to find the point where the likelihood plot leveled off and began to fluctuate around a stable value using Tracer 1.4 [53] (plots not shown). In all cases, the conservative estimate of a burnin of 25% of sampled trees was well into this plateau. The remaining 75000 trees (37500 from each run) were used to construct majority rule consensus trees and calculate posterior probabilities. All clades and support values are shown in the resulting phylogenies. All posterior probabilities are mapped on all trees. As suggested by Hulsenbeck [50], clades with posterior probabilities of 0.95–1.00 will be considered to be very well-supported. Clades with support values of 0.85–0.94 will be considered supported. All posterior probabilities lower than 0.85 are considered poorly supported and should be viewed with caution, but all posterior probabilities are mapped on all trees. Although taxa may appear as sister species, we can only know true sister species relationships if we have complete taxon sampling for the family.

### Nomenclature and Classification

We know from the discovery of polyphyletic and paraphyletic generic groupings in *Chromodoris*, *Glossodoris*, *Hypsleodoris*, *Mexichromis* and *Noumea* [27], [28], that the current classification of the Chromodorididae does not reflect the evolutionary history of the group. We cannot continue to use this current classification. We will use the resulting phylogenies to propose a new classification of the chromodorid nudibranchs. The proposed new classification is based on several fundamental tnets of phylogenetic classification. Only clades are named, with two exceptions described below. Each clade contains the type species of the name-bearing clade. Exisiting, available names are utilized wherever possible to minimize the disruption to nomenclature, while simultaneously reflecting relationship. We will identify clades that include the type species of each chromodorid genus and delineate genera to minimize conflict with current classification and support recognition of interesting morphology.The translation of phylogenetic hypotheses into classifications is the best way to communicate results to a larger community, but even as the number of molecular phylogenies increases, the number of new classifications is decreasing [54]–[56]. The growing phylogeny/classification gap is troubling. Phylogenies are hypotheses of relationship and communicating these new hypotheses is one of the main contributions systematics can make to the scientific community.

Traditional taxonomy has obscured the patterns of diversification in the chromodorids. A new classification that properly reflects evolutionary history is required. In the new classification, we only keep existing names, for species not supported in clades if it is not disruptive to the new classification. We also hypothesize the predicted phylogenetic position of taxa that have not yet been included in the phylogenetic analysis. We used the nomenclatural standards set by the International Code of Zoological Nomenclature [57]. Names and dates for genera, families and subfamilies we taken from Bouchet et al's review of gastropod nomenclature [58]. Every name used is resurrected from synonymy and proposed because the type species of the genus is found in the clade. If more than one generic type species is found in the same clade, the older name has priority. In this way, the history of naming in the chromodorids will be maintained. If the gender of a species' new genus changes in the new classification, the gender of the specific epithet will be changed. Additionally, if the incorrect specific name gender has been used, the proper gender will be used in the new classification. The proposed phylogenetic naming code, the Phylocode, recommends naming clades when type species are part of the clade to be named, as we have done here, but does not use or recognize ranks as we have by using generic, subfamily and family names for clades. In many cases there is no conflict between the phylocode and traditional nomenlclature [59]. The phylocode has not been formally adopted, so there is no official system for naming in accordance with that code. In order to maintain stability and to avoid creating names that may change with the addition of new information, we used a method advocated by Dayrat & Gosliner [60]. This method advocates using the most inclusive known clade name as the first part of a species binomial for species that cannot be named without creating a new name. We will use the family name Chromodorididae as the name for species that would create instability if the bionomials were unchanged or if new names were given. In our proposed classification we will also include incerte sedis species in the Chromodorididae. In clades that are poorly supported (posterior probabilities below 0.85), we have used a generic name for members of those groups with the generic name placed in quotation marks. We prefer this method as an interim solution as it does not leave these taxa in taxonomic limbo and retain the use of single names for polyphyletic groups.

## Results

### Data

The sequenced COI fragment is 658 base pairs (bp) long. The edited 16s sequences are 531 bp long. The combined data sets with gaps introduced for alignment are 1189 base pairs long. All sequences are available from GenBank COI (JQ727822–JQ727914), 16s (JQ727689–JQ727821) and aligned data matrices are available upon request from the corresponding author. Excluded variable 16s regions are identified as character sets in all nexus files. Saturation was not found in the 16s fragment or the first or second positions of the COI fragment. There is slight saturation in the third position transitions in the COI data set (not shown). The third positions were included in the Bayesian analysis as the partitioning allows the parameters of this position to be estimated separately and the inclusion of the third positions did not change the resulting trees. The recommended model of evolution (AIC form Mr.Model Test) was used to set parameters in Mr.Bayes for each partition. The resulting best-fit model of evolution for each partition using the AIC selection from Mr. ModelTest ver.2 [78] were COI 1^st^: GTR+G, COI 2nd: TrN+I+G, COI 3^rd^: GTR+I+G and 16s: GTR+I+G. These models correspond to the following settings in Mr. Bayes; all partitions set to nst = 6 and rates = invgamma except for the COI second codon position partition which was set rates = gamma.

### Phylogeny

The figured trees are the resulting consensus phylograms from the Bayesian analyses (Figures S1, S2). All posterior probabilities are shown above the branches on the Bayesian phylograms. Tree topology was not altered with the inclusion or exclusion or the 16s fragment's variable regions (See Figure S2 for comparison of trees with and without variable regions). The resulting phylogenetic hypotheses for each dataset are summarized below. We will discuss relationships in terms of posterior probabilities.

#### COI and 16s Combined Analysis: Including only specimens with data for both genes (Figure S1)

This data set included 164 individual chromodorids, representing 123 species, three species of Actinocyclidae, four other dorids. The outgroup was *Doris kerguelensis*. The data set included was 1189 bases long included gaps introduced to aid in alignment of variable regions. All bases are included. In the majority rule consensus phylogram resulting from the Bayesian analysis, the chromodorids are monophyletic (pp = 1.00). They are sister (pp = 0.98) to the monophyletic actinocyclids (pp = 1.00). *Cadlinella ornatissima* is sister to the rest of the chromodorids (pp = 1.00). The monophyletic *Tyrinna* (pp = 1.00) is poorly supported as sister to the main clade of chromodorids (pp = 0.82). The main clade of all chromodorids, except *Cadlinella* and *Tyrinna* is supported (pp = 0.85). Two clades of *Noumea* (both pp = 1.00) are part of a basal polytomy with the clade including the remaining chromodorid species (pp = 0.89). A well-supported clade (pp = 1.00) containing some species of *Glossodoris* is poorly supported at the base of the chromodorid grade. Within this main clade of chromodorids, there is one very well supported clade, which includes all species of *Ceratosoma*, *Durvilledoris*, *Hypselodoris*, *Mexichromis*, *Pectenodoris*, *Risbecia*, *Thorunna* and some *Digidentis* (pp = 1.00) *Diversidoris aurantionodulosa* and *Noumea crocea* are sister species (pp = 1.00) and poorly supported (pp = 0.78) as sister to a poorly supported clade (pp = 0.66) that includes this well-supported clade and Chromodoris *alternata* and *Chromodoris ambiguus* (pp = 1.00). There is also a poorly supported clade (or grade if the clade is collapsed) of smaller clades made up of species of *Ardeadoris*, *Chromodoris*, *Diversidoris*, *Glossodoris*, *Noumea*, *Verconia* and one species of *Digidentis* (pp = 0.67). Of the other 12 non-monotypic traditional genera, seven (*Ceratosoma*, *Chromodoris*, *Digidentis*, *Glossodoris*, *Hypselodoris*, *Mexichromis*, *Noumea*) are non-monophyletic and three (*Durvilledoris*, *Pectenodoris*, *Risbecia*) are monophyletic but render another genus paraphyletic. Both *Ardeadoris* and *Thorunna* are made paraphyletic by nested members of other genera (*Noumea*, *Glossodoris*-within Ardeadoris and *Digidentis*-within *Thorunna*). There are three species and five clades of eastern Pacific and/or Atlantic species.

#### COI and 16s Combined Analysis: Including all specimens (Figure S2)

This data set included 244 individual chromodorids, representing 157 species, four species of Actinocyclidae, four other dorids. The outgroup was *Doris kerguelensis*.

**Figure 3:**
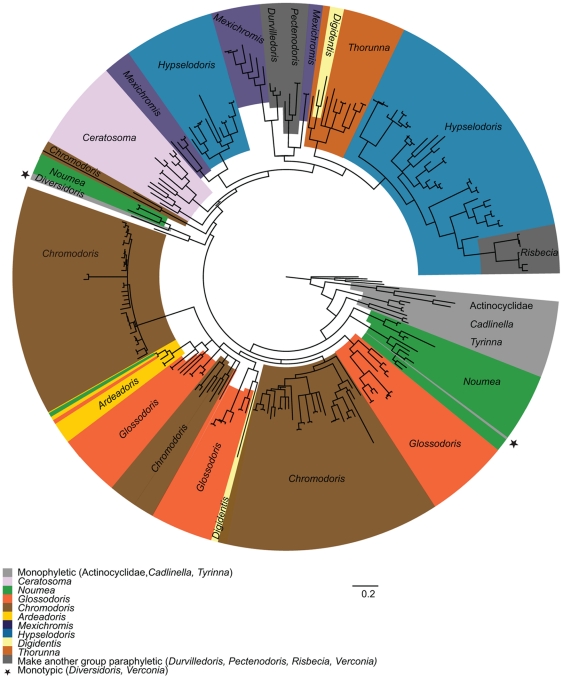
Circle phylogram with current generic names. Tree is the same Bayesian phylogram as figured in S3A. All specimens, both genes and all characters included.

**Figure 4:**
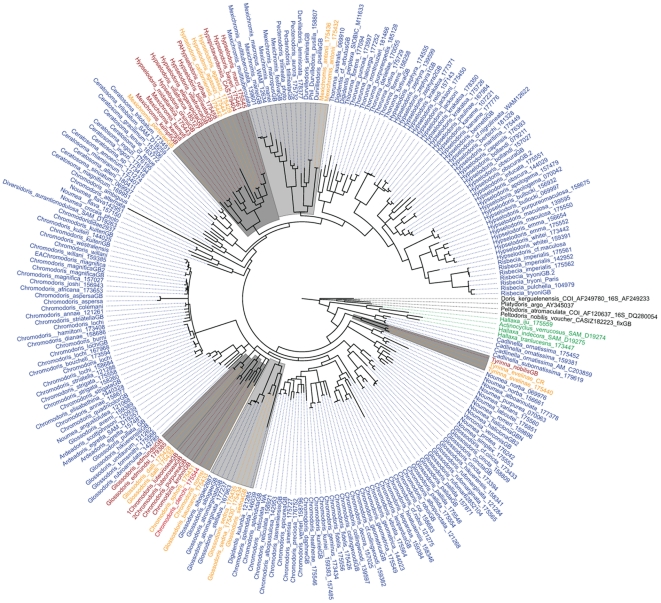
Biogeography mapped on circle phylogram. Tree is the same Bayesian phylogram as figured in S3A. All specimens, both genes and all characters included. Blue = Indo-Pacific, Red = Atlantic and Caribbean, Gold = Eastern Pacific, Green = Sister group, Black = outgroups. Dark grey = Solely eastern Pacific and Atlantic clades. Light grey = Primarily Indo-Pacific clades with eastern Pacific members.

The complete data set included 1189 bases. The chromodorids are monophyletic (pp = 0.94). They are sister to the monophyletic actinocyclids (pp = 0.98). A clade including both species of *Cadlinella* is sister to the rest of the chromodorids (pp = 1.00). The monophyletic *Tyrinna* (pp = 1.00) is poorly supported as sister to the main clade (pp = 0.83). There are two clades containing species of *Noumea* and the one species of *Verconia* (pp = 1.00 and pp = 1.00) that form a polytomy with the clade of all of the remaining chromodorids (pp = 0.85). Within the main clade of chromodorids, there is one very well supported clade, which includes all species of *Ceratosoma*, *Durvilledoris*, *Hypselodoris*, *Mexichromis*, *Pectenodoris*, *Risbecia*, *Thorunna* and some *Digidentis* (pp = 1.00) and a grade of clades of species *Ardeadoris*, *Chromodoris*, *Diversidoris*, *Glossodoris*, *Noumea*, *Verconia* and one species of *Digidentis*. Of the other 12 non-monotypic traditional genera, seven (*Ceratosoma*, *Chromodoris*, *Digidentis*, *Glossodoris*, *Hypselodoris*, *Mexichromis*, *Noumea*) are non-monophyletic and three (*Durvilledoris*, *Pectenodoris*, *Risbecia*) are monophyletic but render another genus paraphyletic. Both *Ardeadoris* and *Thorunna* are made paraphyletic by nested members of other genera (*Noumea*, *Glossodoris* and *Digidentis*) (Figure 3). More detailed results found within each clade will be discussed below. There are three individual species and five clades of eastern Pacific or Atlantic species (Figure 4). The data set without variable regions included 1108 bases. There are only slight changes to the tree topology, including slight losses of support and changes to branching pattern in the species relationships within four clades containing species of *Noumea*, *Glossodoris*, *Chromodoris* and *Thorunna*. Additionally, some clades are more well-supported in the phylogram without variable regions, most notably there is some support for the two *Noumea* clades as sisters. All of these differences are mapped in mirrored trees (Figure S2).

## Discussion

### New Classification of the Chromodorididae (Table S2)

**Figure 5:**
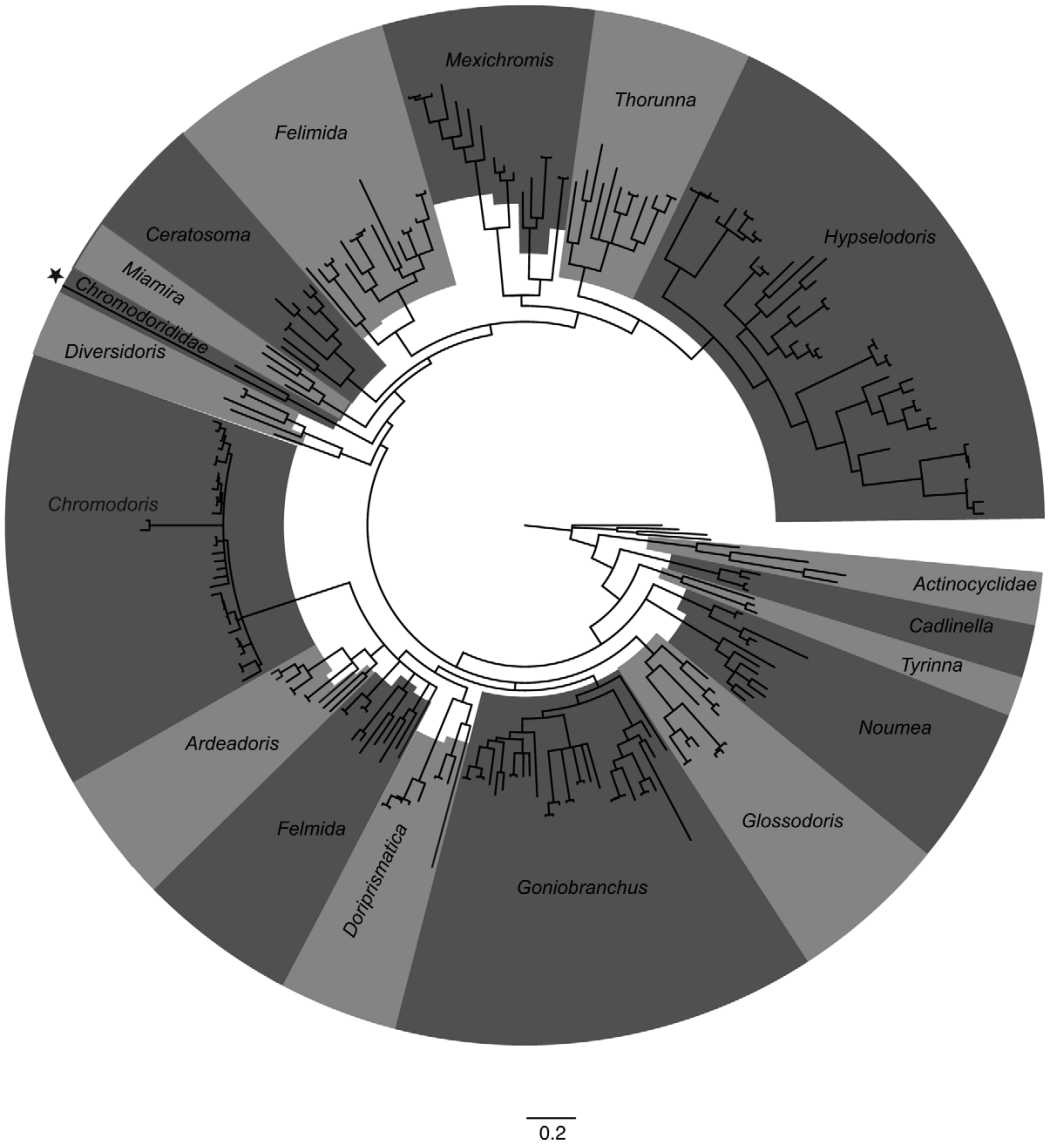
Circle phylogram with new generic and clade names. Tree is the same Bayesian phylogram as figured in S3A. All specimens, both genes and all characters included.

Our classification is based on our COI and 16s combined phylogeny with all specimens included (Figure S2A). The older name that describes this clade, Ceratosomatidae Gray 1857 [61] was declared *nomen oblitum* under Art. 23.9 of the International Code of Zoological Nomenclature [58]. Even though it is older than the name in current usage, Chromodorididae, it had not been used in over fifty years. In the phylogeny of the chromodorid nudibranchs, there are five basal clades: *Cadlinella*, *Tyrinna*, two clades made up of some species of *Noumea* and *Verconia* and one clade made up of some species of *Glossodoris*. There is one, main, well-supported clade including species of *Ceratosoma*, *Hypselodoris*, and grade of clades. We will briefly introduce each clade and its member species in the context of a new classification for chromodorid nudibranchs (Figure 5, S2A, S3).

Of the 16 genera in the current chromodorid classficiation, only two, *Cadlinella* and *Tyrinna*, retain the same membership in our new classification. Nine more generic names are retained; *Ardeadoris*, *Ceratosoma*, *Chromodoris*, *Diversidoris*, *Glossodoris*, *Hypselodoris*, *Mexichromis*, *Noumea* and *Thorunna* but their membership has changed. Five names are synonymized with the names listed above: *Digidentis*, *Durvilledoris*, *Pectenodoris*, *Risbecia* and *Verconia*. Five older names: *Doriprismatica*, *Felimare*, *Felimida*, *Goniobranchus* and *Miamira*, have been rescued from synonymy and are used to describe clades of species previously included in different genera. There are 17 generic names used in our new classification, each of which will be detailed below and in Figure S3. Of these, 13 are very well supported with posterior probabilities ≥0.95. *Mexichromis* is supported with a posterior probability of 0.87 when variable positions are included and 0.95 when they are excluded. *Noumea* consists of two separate clades (both pp = 1.00) that are poorly supported as a combined clade in the analysis when variable positions are included (pp = 0.61). Although, this support is not sufficient, all of the species in both of these clades are currently named *Noumea* and will retain this name in order to maintain stability. *Doriprismatica* is extremely poorly-supported pp = 0.64 with variable positions included and well-supported (pp = 0.92) in the analysis with variable positions excluded (Figures S2, S3). We do not consider this level of support sufficient to definitively name this clade, but because continuing to use the current name, *Chromodoris*, would add greater confusion (as that name represents a different, well-supported monophyletic group) we will premilinarily name these species ‘*Doriprismatica’*. Similarly, a group of species some of which are currently classified as *Chromodoris* and some as *Glossodoris* form a polytomy together with other well supported clades. As these species need a name, but lack appropriate support, they cannot be named definitively. We will preliminaryily name these species *‘Felimida’* . Naming these clades is much more stable than using names that now represent other well-defined and well supported clades. These names are hypotheses; with more data the relationships of members of these clades will likely become better resolved. The completed classisifiction is listed in Table S2.

### Chromodorididae Bergh 1891 [84]

#### *Cadlinella* Thiele, 1931 [62]

Type species: *Cadlina ornatissima* Risbec, 1928 [63] (by monotypy)

The two species of *Cadlinella* included here, *Cadlinella ornatissima* and *Cadlinellla subornatissima* form a clade and are sister to the rest of the chromodorid species (pp = 1.00). These findings support previous results [27], [28] and Rudman's evolutionary scenario [5], [26]. The widespread Indo-Pacific genus, *Cadlinella* is an enigmatic taxon. It has at different times been considered it own separate family [64], a part of the Cadlininae [65] and a member of the Chromodoridinae/Chromodorididae [26], [66].

#### *Tyrinna* Bergh, 1898 [67]

Type species: *Tyrinna nobilis* Bergh, 1898 [67] (by monotypy)

Synonymy

*Cadlina burnayi* Ortea, 1988 [68] = T. nobilis [69]

The only two species of *Tyrinna*: *T. evelinae* and *T. nobilis* are included here. *Tyrinna* is always monophyletic (pp = 1.00). After the split from *Cadlinella*, this clade is poorly-supported as the sister group to the main group of chromodorids (pp = 0.83). Rudman [26] suggested that *Tyrinna*, *Cadlinella* and *Cadlina* form a basal grade of primitive chromodorids. *Cadlina* had been shown not to be a chromodorid [28], but our results support Rudman's suggestion that *Tyrinna* and *Cadlinella* are basal to the rest of the chromodorids. Muniain et al [70] and Schrödl and Millen [71] extensively reviewed the morphology of the two species in this clade.

#### *Noumea* Risbec, 1928 [63]

Type species: *Noumea romeri* (by subsequent designation Baba, 1937 [72])

Synonymy

*Verconia* Pruvot-Fol, 1931 [73]

Type species: *Albania*? *verconis* Basedow and Hedley, 1905 [74] (by monotypy)

*Verconia verconis* is well supported as part of a clade that includes *N. haliclona*, *N. laboutei*, *N.romeri* and *N. simplex* (pp = 1.00). *Noumea varians*, *N. purpurea* and *N. norba* form a well-supported clade (pp = 1.00) that is not part of a name bearing clade, but is one branch of the polytomy that includes the *‘Noumea sensu stricto’* and the branch leading to the rest of the family (pp = 0.88). The monotypic genus *Verconia* is nested within the *Noumea* clade as suggested by Rudman [75] and weakly supported as the sister species to another South Australian species, *N. haliclona*, as found in the preliminary results shown by Turner & Wilson [27].

#### *Glossodoris* Ehrenberg, 1831 [76]

*Type species: Doris xantholeuca Ehrenberg, 1831* [*76*] *= G. pallida (by subsequent designation)*

The *Glossodoris* clade (pp = 1.00) includes species *G. pallida* and *G. rufomargninata*. In an important, but often overlooked detailed examination of the relationships of the species classified in the genus *Glossodoris*, Rudman identified five subgroups of this genus based on morphology [77]. The species in this *Glossodoris* clade were considered by Rudman [77] to be members of the ‘*Glossodoris pallida* subgroup’. This clade also includes two species he did not include in any subgroup, *G. cincta* and *G. hikuerenesis*.

#### *Goniobranchus* Pease, 1866 [78]

Type species: *Goniobranchus vibrata* Pease, 1866 [78] (by subsequent designation)

Synonymy

*Lissodoris* Odhner, 1934 [80]. Type species: *L. mollis* Odhner, 1934 ( = *C. aureomarginata* Cheeseman, 1881[86] (by monotypy)

This clade includes all of the Indo-Pacific species of *Chromodoris* that are not part of the black-lined, planar egg mass clade (pp = 1.00), except *Chromodoris alternata* and *Chromodoris ambiguus*. This phylogeny is the first to find definitive support for a clade of chromodorids, first suggested by Wilson [16] and Turner and Wilson [27] known to lay egg masses with extra-capsular yolk. When Pease designated *Doris vibrata* as the type species for the new genus *Goniobranchus*, he should have changed the ending of *vibrata* to *vibratus* to reflect the masculine gender of the –us ending. We have made that correction here and changed the gender of all of the species names that require changing (names derived from adjectives) in *Goniobranchus*.

#### *‘Doriprismatica’* d'Orbigny, 1839 [81]

Type species: *Doris atromarginata* Cuvier, 1804 [82] (by subsequent designation-Herrmannsen, 1847[83])

Synonymy

*Casella* H. & A. Adams, 1858:57 [84]. Type species: *C. gouldii* H. & A. Adams, 1858 [84] (by monotypy)

*Chromolaichma* Bertsch, 1977: 113 [65]. Type species: *Casella sedna* Marcus & Marcus, 1967 [85] (by original designation)

Species included in the *Glossodoris atromarginata* subgroup [77] are recovered in this clade, with the addition of *G. sedna* and *Digidentis kulonba* (pp = 0.95).

#### *‘Felimida’* Marcus, 1971 [86]

*Type species: Felimida sphoni Marcus, 1971* [*86*] *(by monotypy)*

This name will be used for all eastern Pacific and Atlantic species of *Chromodoris* and *Glossodoris* (except *Glossodoris sedna*). These species form a polytomy including *Glossodoris baumanni* and three clades of Atlantic and Eastern Pacific chromodorids.

Chromodoris clenchi, C. norrisi and C. sphoni (pp = 1.00)

Glossodoris dalli and G. edmundsi (pp = 1.00)

Chromodoris krohni, C. luteorosea and C. purpurea (pp = 0.78)

These exclusively eastern Pacific and Atlantic clades do not form a monophyletic group, but we will provisionally name all of these species *‘Felimida’*. This is the most conservative choice, the choice that requires the fewest name changes and is the least disruptive pending further information and broader taxon sampling.

#### *Ardeadoris* Rudman, 1984 [26]

Type species: *Ardeadoris egretta* Rudman, 1984 [26] (by original designation)

The Ardeadoris clade includes both species of Ardeadoris: A. egretta and A. scottjohnsoni, five species of Glossodoris (G. averni, G. pullata, G. rubroannulata, G. tomsmithi and Glossodoris undaurum) and Noumea angustolutea (pp = 1.00). . Based on their analysis, Turner and Wilson [27] suggested that with more sampling it would be come clear if Ardeadoris should be synonmized with Glossodoris. By sampling more broadly within the family, we found the converse. Four species of Glossodoris and Noumea angustolutea need to be included within Ardeardoris because they are strongly supported as part of the clade including Ardeadoris egretta and not the type species of Glossodoris. Three of the species, G. averni, G. undaurum and G. rubroannulata, found in this clade were part of Rudman's Glossodoris sedna subgroup [77]

#### *Chromodoris* Alder & Hanncock, 1855 [87]

Type species: *Doris magnifica* Quoy and Gaimard, 1832 [88] (by original designation)

This clade includes all of the black-lined species *of Chromodoris and Chromodoris aspersa* (pp = 1.00). This clade was identified by, both Wilson & Lee [17] and Turner & Wilson [27], as the planar spawning or black-lined *Chromodoris* clade. All of the members of this clade lay flat egg masses.

#### *Diversidoris* Rudman, 1987 [89]

*Type species: Diversidoris aurantionodulosa Rudman, 1987* [*89*] *(by original designation).*

The *Diversidoris* includes, *Diversidoris aurantionodulosa*, two yellow species of *Noumea*, *N. crocea* and *N. flava*, and a new species from Moorea, French Polynesia-Chromodoridae BioCode 2937 (pp = 0.95).

### Miamirinae Bergh 1891 [90]

The Miamirinae clade includes all of the species currently classified as *Ceratosoma*, *Durvilledoris*, *Hypselodoris*, *Mexichromis*, *Pectenodoris*, *Risbecia*, *Thorunna* and two species of *Digidentis* (pp = 1.00)

Remarks

This clade was first predicted by Rudman [26] based on morphological similarities and then confirmed by Rudman & Berquist's [5] finding that all of the species in this clade feed exclusively on sponges of the family Dysideaidae, although they assumed all of the genera to be monophyletic. Miamirinae Bergh 1891 is the oldest appropriate and available subfamily or family name for this clade. The remaining six genera; *Miamira, Ceratosoma, Felimare, Mexichromis, Thorunna* and *Hypselodoris* make up the Miamirinae.

#### *Miamira* Bergh, 1874 [91]

Type species: *Miamira nobilis* Bergh, 1874 [91] (by monotypy)

Synonymy

*Orodoris* Bergh, 1875 [92]. Type species: *Orodoris miamirana* Bergh, 1875 [92] (by monotypy)

The *Miamira* clade includes the following species (as currently classified) *Ceratosoma alleni*, *Ceratosoma magnificum, Ceratosoma miamiranum, Ceratosoma sinuatum*. *Miamira* is part of a grade with *Ceratosoma*. The morphological phylogeny of species of *Ceratosoma* and classified as *Miamira* and *Orodoris*, that was used as justification for their synonomy, predicted a sister group relationship between species of *Miamira* and *Ceratosoma alleni* [93]. Our results confirm that *C. alleni* is more closely related to species of *Miamira*, but do not find support for synonymy of *Miamira* and *Ceratosoma*. Although, it is possible this relationship will be recovered with further sampling and by including molecular markers that will help resolve basal branches on the phylogeny.

#### *Ceratosoma* Adams and Reeve, 1850 [94]

Type species: *Ceratosoma cornigerum* Adam and Reeve, 1850 [94] (by monotypy)

The *Ceratosoma* clade includes *C. amoenum, C. gracillimum, C. ingozi, C. tenue, C. trilobatum* and a new species. (pp = 1.00)

#### *Felimare* Marcus and Marcus, 1967 [85]

Type species: *Felimare bayeri* Marcus and Marcus, 1967 [85] (by monotypy)

The *Felimare* clade includes all eastern Pacific, Atlantic and Mediterranean species of *Hypselodoris* and two species of *Mexichromis, M. porterae* and *M. kempfi* from the eastern Pacific and Caribbean respectively (pp = 1.00). Both Gosliner and Johnson [13] and Alejandrino and Valdés [31] hypothesized a sister group relationship between the Indo-Pacific and eastern Pacific/Atlantic species of *Hypselodoris*. Turner and Wilson [27] did not recover that relationship, but instead found the same relationships shown here.

#### *Mexichromis* Bertsch, 1977 [65]

Type species: *Chromodoris antonii* Bertsch, 1976 [95] (by original designation)

Synonymy

*Durvilledoris* Rudman, 1984 [26]. Type species: *Doris lemniscata* Quoy and Gaimard, 1832 [88] (by original designation)

*Pectenodoris* Rudman, 1984 [26]. Type species: *Goniodoris trilineata* Adams & Reeve, 1850 [94] (by original designation)

This clade includes the type species of *Mexichromis*, *M. antonii*, known only from the eastern Pacific and the three included species of *Durvilledoris, D. lemniscata, D. pusilla* and *D. similaris*, the two described species of *Pectenodoris, P. aurora* and *P. trilineata* and all of the Indo-Pacific species currently considered *Mexichromis, M. festiva, M. macropus, M. mariei* and *M. mutituberculata* (pp = 1.00). There are two well-supported clades within the *Mexichromis* clade. The clade including *Mexichromis antonii* and the species of *Durvilledoris* is sister to the clade including *Pectenodoris* and Indo- Pacific *Mexichromis*. These clades could be given two names, but it is much less disruptive and confusing to maintain the name *Mexichromis* for all clade members. The clade including P. *aurora* and *P. trilineata* can be called the ‘*Pectenodoris*’ clade of *Mexichromis*.

#### *Thorunna* Bergh, 1878 [96]

Type species: *Thorunna furtiva* Bergh, 1878 [96] (by monotypy)

Synonymy

*Digidentis* Rudman, 1984 [26]. Type species: *Glossodoris arbuta* Burn, 1961 [97] (by original designation)

The *Thorunna* clade includes all species of *Thorunna* and two species of *Digidentis*, *D. arbutus* and *D. perplexa*. All of species currently classified as *Thorunna* are found in the Indo-Pacific and the species of *Digidentis* are limited to southern Australia. As suggested by Rudman [26], the only species within *Thorunna* with mantle glands, *T. australis* and the species of *Digidentis* (all of which have mantle glands) form a clade.

#### *Hypselodoris* Stimpson, 1855 [98]

Type species: *Goniodoris obscura* Stimpson, 1855 [98] (by monotypy)

Synonymy

*Risbecia* Odhner, 1934 [79]. Type species: *Ceratosoma francoisi* Risbec, 1928 [63] (by original designation)

This clade includes all of the Indo-Pacific species of *Hypeslodoris* and *Risbecia* (pp = 1.00).

Species of *Risbecia s.s* forms a well-supported clade nested within *Hypselodoris* and can be referred to as the Risbecia clade of *Hypselodoris*. *Risbecia aplogema* is not part of this *Risbecia* clade and was previously considered a species of *Hypselodoris*. Including all of the members of the *Risbecia* and *Hypselodoris bullocki* clade in *Risbecia* is not an option because this would render *Hypsleodoris* paraphyletic. The second clade includes, *H. bennetti, H, maritima, H. bertschi, H. paulinae, H. kaname, H. bollandi, H. obscura, H. infucata, H. zephrya* and one or two new species. The third clade includes *H. reidi, H. krakatoa, H. jacksoni* and one new species. This clade was also recovered in Gosliner & Johnson's [13] morphological phylogeny of *Hypselodoris*.

### Chromodorididae

#### *Chromodoris alternata* and *Chromodoris ambiguus*

The enigmatic south Australian species, *Chromodoris alternata* and *C. ambiguus* are very different than other chromodorids. They are two of the five chromodorid species with a plesiomorphic serial reproductive system (*C. loringi, C.thompsoni, C. woddwardae*) [26], [28], [89]. All five of these species are found only in southeastern Australia. These species were found to be more closely related to *Cadlina* than *Chromodoris* by Wilson & Lee [17], but as part of the chromodorid grade in Turner & Wilson [27]. Clearly further work on this group and its relationship to all cryptobranchs is needed. The addition of specimens of *C. loringi*, *C. thompsoni* and *C. woodwardae* [26], [89], [99], the only other chromodorid species known to have a serial reproductive system may help solve this problem. These two species are always each other's closest relatives and are sister to the rest of the Miamirainae in the all analyses. As suggested by Dayrat & Gosliner [60] they should be considered Chromodorididae, because they are not included in a named clade. Until the ambiguity of the relationship of these taxa to other chromodorids can be resolved, they should be considered Chromodorididae *alternata* and Chromodorididae *ambiguous*.

#### Phylogeny of the chromodorid nudibranchs

The primary goal of this study was to generate a phylogeny of the chromodorid nudibranchs and present a classification that accurately reflects the evolutionary history of this group. We have included mitochondrial DNA sequence data for 157 chromodorid species, more than double previous sampling and making this the largest species level phylogeny of nudibranchs ever published. We included the type species of every genus; complete sampling of every described species for five of the sixteen genera (*Ardeadoris, Diversidoris, Pectenodoris*, *Tyrinna* and *Verconia*) and more than half of the species in every other genus. With this sampling, we were able to test the monophyly of all the chromodorid genera. Both Rudman's ‘*Chromodoris* subgroup’ including species of *Ardeadoris, Chromodoris, Glossodoris, Noumea* (only some species, other species currently considered *Noumea* are also found in the *Ardeadoris* and *Diversidoris* clades) and his ‘*Hypselodoris* subgroup’ with species of *Ceratosoma, Hypselodoris, Mexichromis* and *Thorunna* were recovered. Although these subgroups can be observed, none of the chromodorid genera is monophyletic, except *Cadlinella* and *Tyrinna* (see above). In every case, a genus either is polyphyletic or it is nested within another genus; and therefore it makes another genus paraphyletic. This result at once illuminates both the difficulty of delineating natural groups in very diverse and homoplastic clades and the insight that can be gained from systematic reviews, like Rudman's [26] review of the family. Rudman [26] was able to discern the two main groups of chromodorids without a phylogenetic analysis or molecular data. At a large enough scale, it is possible to sort out synapomorphies, but on a smaller scale, homoplasy muddies the waters. The problem of homoplasy confusing taxonomy and systematics in nudibranchs has been explored [100], but is not generally mentioned in the description of new genera. This is largely a result of the fact that the majority of new taxa have been described without a phylogenetic hypotheses and similar morphology is rarely discussed outside the possibility of close relationship rather than being a result of homoplasy. Traditionally, new genera were often erected on the basis of a single evolutionary novelty. Many of thes attributes prove to be autapomorphies in these taxa and do not consitiute a basis for establishing these genera as clades when subjected to phylogenetic analysis.

In future contribtuions, we will work out synapomorphies for the clades identified here, but because of the amount of homoplasy and number of incomplete descriptions, this is a huge undertaking and not appropriate here.

#### Monophyly of the Chromodorididae

Bergh [90] was the first to suggest a separate taxonomic rank for the chromodorid nudibranchs. Johnson [28] showed that the Chromodorididae are only monophyletic if *Cadlina* is removed from the family, as *Cadlina* is more closely related to *Aldisa* and other dorid nudibranchs. We expanded on these preliminary results and confirmed the monophyly of the chromodorids in analyses without *Cadlina* and without including as many dorid species. Gosliner and Johnson [101] reviewed the genus *Hallaxa* and presented a morphological phylogeny of *Hallaxa* and *Actinocylcus*. They hypothesized that the semi-serial reproductive system found in species of *Hallaxa* and *Actinocyclus* and all chromodorids is a synapomorphy that unites the two groups. The chromodorid nudibranchs are monophyletic in every analysis, as is their sister group relationship with Actinocyclidae. These analyses confirm the hypothesis of Gosliner & Johnson [101] and the preliminary findings of Johnson [28] and confirm the utility of this morphological synapomorphy.

#### Chromodorid phylogeny

Most previous work has assumed monophyly of chromodorid genera; subsequently work on the natural history of chromodorids has used genera as *de facto* hypotheses of relationship. Genera should only be used in this way if they are known to be monophyletic through phylogenetic analysis. There have been two classes of ‘naming problems’ in the nomenclatural history of the chromodorid nudibranchs. The first can be described as the novelty problem (as described above), when unique or ‘unclassifiable’ species were discovered new genera we created to contain them [26], [89]. And the second, the ‘catch-all’ problem, new species were assigned to large genera with the widest definitions [60]. Considering these genera as evolutionary units at the broad scale may not lead to mistakes, but at a finer scale, we may be missing the true origins of novelty or by grouping things that are superficially similar together we may miss repeated origins of diversity (convergence). Turner & Wilson [27] were the first to truly test generic monophyly in more than one chromodorid genus (See Figure 1 for previous phylogenetic hypotheses). They found evidence for the non-monophyly of most chromodorid genera. The only genera they found to be monophyletic were *Digidentis* (pp = 1.00), and *Durvilledoris* (pp = 1.00). Of the genera they could test, they found *Chromodoris, Glossodoris, Hypselodoris* and *Mexichromis* to be paraphyletic or polyphyletic. They also found *Risbecia* to be monophyletic, but nested within *Hypselodoris*. They used their findings as evidence for the ‘polyphyly of widespread genera’ or species currently classified in different genera found in one ocean basin more closely related to each other than to their congeners found in other oceans. For example, they found *Mexichromis porterae*, a species known only from the eastern Pacific, to be more closely related to species of *Hypselodoris* from the eastern Pacific than either were to *Mexichromis* or *Hypsleodoris* species found in the Indo-Pacific. This finding tells us something new, but it actually does not tell us much about biogeography, because the genera they discussed were not monophyltic entities. They uncovered a taxonomic problem, not a biogeographic or biological one. Their results actually confirm the taxonomic confusion the authors of most of the species they sampled expressed when faced with choosing a generic placement for new taxa. In fact, all of these authors established new genera to account for differences they found, *Mexichromis*, *Felimare*, *Felimida*, *Digidentis*, *Ardeadoris*, *Durvilledoris* etc [26], [65], [85], [86]. It was primarily by changes to the generic placement by subsequent authors, synonymy of, and the addition of taxa to, newly created genera that lead to the ‘polyphyly across the oceans’ [26], [102]. The phylogeny presented here allows informed exploration of the taxonomic and nomenclatural history of the chromodorid nudibranchs. The clades we recovered in this molecular phylogeny are even more interesting when we use the taxonomic history, in the form of older generic names attached to type species, as a map of the discovery of the great diversity of this group. Our studies indicate that when monophyletic units are recognized, there is strong biogeographical signal rather than “polyphyly across oceans”.

In summary, with the most comprehensive sampling of chromodorid species to date, we confirmed that the chromodorids are monophyletic and are sister to the monophyletic actinocyclids. We also found that the majority, 12/14 non-monotypic traditional genera, were not monophyletic or make another clade paraphyletc. Seven traditional genera, *Ceratosoma*, *Chromodoris*, *Digidentis*, *Glossodoris*, *Hypselodoris*, *Mexichromis*, *Noumea* are non-monophyletic and three (*Durvilledoris*, *Pectenodoris*, *Risbecia*) are monophyletic but render another genus paraphyletic. Both *Ardeadoris* and *Thorunna* are made paraphyletic by nested members of other genera (*Noumea*, *Glossodoris* and *Digidentis*). The two monotypic genera, *Diversidoris* and *Verconia* are nested within clades. Only *Tyrinna* and *Cadlinella* are monophyletic and without disruption to any other clades (Figure 3, S1, S2). The classification proposed here and discussed at length above renames clades and is more consisitent with evolutionary history (Figure S3).

#### Biogeography in light of new classification

The most speciose chromodorid genera: *Chromodoris*, *Glossodoris* and *Hypselodoris* were originally created to describe Indo-Pacific species. It wasn't until some time after these names were created that previously described, similar, brightly colored cryptobranch dorid species found in the eastern Pacific, western Atlantic and Mediterranean were added to these genera [1], [26], [65], [95], [102], [103]. In *Mexichromis* the opposite is true. The type species, *Mexichromis antonii*, was described from the eastern Pacific and Indo-Pacific species were included later included in this genus [26], [95]. Other eastern Pacific “*Mexichromis*” are shown here to belong to *Felimare*.

This new classification clarifies our view of biogeographic patterns in the chromodorid nudibranchs. Instead of taxonomy obscuring patterns of diversification in this group, this taxonomy reflects and reinforces evolutionary history. It gives us a much better framework for exploring evolutionary questions.

The majority of chromodorid nudibranchs are found in the Indo-Pacific, but there are three individual species and five clades of solely Atlantic and/or eastern Pacific species (Figure 4). The sister group to the rest of the chromodorids, *Cadlinella* is found only in the Indo-Pacific, while the sister to the Chromodorididae, the Actinocyclidae is found in most temperate and tropical waters. Although there are other possibly scenarios, such as trans-Pacific dispersal and migration around Africa, the pattern uncovered here, strongly supports the simplest hypothesis that the chromodorids diversified rapidly from the tropical Tethyan Realm. This pattern has been found in other gastropod groups [104]–[108] (Figure 4). The chromodorids were likely widely distributed and different lineages diversified in isolation following vicariant events. This scenario is further supported by the fact that *Goniobranchis* is sister to ‘*Doriprismatica*’ and its closest realitves and that all the memebers of *Goniobranchus* are Indo-Pacific. Also in this scenario, the specimens identified as *D. sedna* from the Atlantic and eastern Pacific appear to be distinct species as indicated by COI pairwise distances of 11.7–11.0% between eastern Pacific and Altantic specimens while the three eastern Pacific specimens are 0–0.7% different from each other. This scenario clearly supports vicariance between the Indo-Pacific and eastern Pacific and Atlantic preceding the vicariance between the eastern Pacific and Atlantic. In the main chromodorid grade of clades there are two individual species and three clades that are exclusively Atlantic and/or eastern Pacific. Specimens identified as ‘*Doriprismatica’ sedna* found both in the eastern Pacific and the western Atlantic, are always sisters and are nested within a clade of exclusively Indo-Pacific species. This is most likely a radiation into the eastern Pacific and Atlantic from the Indo-Pacific. The remainder of the Atlantic and eastern Pacific species, not included in the Miamirinae, are part of a polytomy including five clades, three containing only eastern Pacific and Atlantic species of *‘Felimida’* and the Indo- Pacific *Ardeadoris* and *Chromodoris* clades. *‘Felimida’ baumanni*, found in the eastern Pacific, is also part of this polytomy. The relationships in this grade need to be examined more closely with the addition of more specimens and more genes.

Relationships within the Miramirinae clade are more resolved. There are two clades that include eastern Pacific and Atlantic species. The *Felimare* clade is exclusively eastern Pacific and Atlantic. There are two eastern Pacific and Atlantic splits in this clade, the eastern Pacific *F. porterae* and Caribbean *F. kempfi* are potentially geminate species and are sister to a larger clade of eastern Pacific, Caribbean and eastern Atlantic *Felimare* species. More sampling is needed in this clade to further untangle the emergent biogeographic patterns within *Felimare*. Additionally, within the Miamirinae, the eastern Pacific species, *Mexichromis antonii*, is sister to the exclusively Indo-Pacific *M. lemniscata*, *M. pusilla* and *M. similaris* and this clade is sister to the rest of the Indo- Pacific *Mexichromis*. Within the Miamirinae, it appears that there had been more than one dispersal event from the Indo-Pacific, to eastern Pacific and Atlantic. *Ceratosoma* and *Miamira* species are only found in the Indo-Pacific and adjacent temperate regions. The sister taxon, the clade including: *Felimare*, *Mexichromis*, *Thorunna* and *Hypselodoris*, has a wider distribution. Within this sister taxon, *Felilmare* is exclusively eastern Pacific and Atlantic while its sister species in its sister taxon *Mexichromis*, *Thorunna* and *Hypselodoris* are almost exclusively Indo-Pacific and adjacent temperate regions. *Mexichromis antonii*, which is found in the eastern Pacific, is the only speices in this clade not found in the Indo-Pacific or adjacent regions. Thus, *Felimare* and *Mexichromis antonii* represent two distrinct invasions of the eastern Pacific from the Indo-Pacific.

#### Future work

We hope the work presented here will serve as a starting point for further research into the evolutionary history of the chromodorid nudibranchs. This phylogeny is based only on mitochondrial genes, one of our first next steps will be to include sequences from nuclear genes for all of the species included here. The addition of more slowly evolving unlinked markers should help resolve some poorly supported node at the base of the phylogenies presented here and will add a separate line of evidence to this hypothesis. Addtionally, morphological synapomorphies need to be found for the clades recoverd in this phyogeny. There is still much work to do in order to untangle the evolutionary history of this group. This phylogeny and classification is a start that will allow us to use names that represent monophyetic groups as the starting point for future discovery.

#### Conclusion

The resulting classification can be used to address questions of interest to a much broader community. A robust phylogeny and corresponding revised classification are necessary to conduct comparative studies in the Chromodorididae. Evolutionary studies of trophic specialization, color patterns and secondary metabolites, for example, will be much more robust by comparing monophyletic units that are clearly named within a new classification. We have pointed out and highlighted many areas of future research. As we detail above, there are many taxonomic, nomenclatural and species delineation problems that still require refinement within the chromodorid nudibranchs. These questions can best be answered with a detailed examination of morphology together with molecular data. The molecular data needs to be rooted in sound identifications and definitions that are always based on morphology. This phylogeny does not answer all of these issues but it will serve as a framework to more effectively tackle these questions. Our classification will serve as a more refined basis for other evolutionary biologists, ecologists and natural products chemists. Their results will be more informative in light of a classification based on evolutionary history rather than one based on untested hypotheses. Phylogenetic systematics provides a rigorous and repeatable methodology that permits iterative approximations of relationship and our understanding of this diverse and biologically intriguing group of organisms is enhanced by these studies.

### Nomenclature

The electronic version of this document does not represent a published work according to the International Code of Zoological Nomenclature (ICZN), and hence the nomenclatural acts contained in the electronic version are not available under that Code from the electronic edition. Therefore, a separate edition of this document was produced by a method that assures numerous identical and durable copies, and those copies were simultaneously obtainable (from the publication date noted on the first page of this article) for the purpose of providing a public and permanent scientific record, in accordance with Article 8.1 of the Code. The separate print-only edition is available on request from PLoS by sending a request to PLoS ONE, Public Library of Science, 1160 Battery Street, Suite 100, San Francisco, CA 94111, USA along with a check for $10 (to cover printing and postage) payable to “Public Library of Science”.

## Supporting Information

Table S1 **Table of Specimens Used in this Study.** Specimens used in this study listed by Family. The names in this table reflect current classification not proposed classification (new names are listed in the text). Abbreviations are as follows: CASIZ = California Academy of Sciences, SAM = South Australian Museum, WAM = Western Australian Museum, AM = Australian Museum, ZSM = Zoologische Staatssammlung München, SIO-BIC = Scripps Institute of Oceanography, BioCode = Moorea BioCode Project.
(DOCX)

Table S2 **New Classification of the Chromodorididae with synonyms.** Generic names and type species in bold and the most recent genus membership follows. Listing order follows phylogeny.
(DOCX)

Figure S1 **Bayesian consensus phylogram including all specimens with data for both genes.** Posterior probabilities are listed above branches. *Doris kerguelensis* is the outgroup. This phylogram is the consensus of 50,000,000 generations with trees sampled every 1000 generations with a burnin of 25%. Data was partitioned by gene and by codon position.
(EPS)

Figure S2 **Bayesian consensus phylograms including all specimens.** A. Phylogram resulting from the inclusion of all characters B. Phylogram resulting from excluding hard to align characters. *Doris kerguelensis* is the outgroup. These phylograms are the consensuses of 50,000,000 generations with trees sampled every 1000 generations with a burnin of 25%. Data was partitioned by gene and by codon position. Dotted lined indicate areas of disagreement.
(EPS)

Figure S3 **Cladogram drawn from Bayesian tree with all specimens, both genes and all characters included.** (S3A). Posterior probabilities are listed above branches. New generic names are used in the tree. Types are in **bold**. Photos of selected specimens are for reference and to show the range of diversity in each genus and the family. From top to bottom: ***Cadlinella*** **Thiele, 1931** *Cadlinella ornatissima*, CASIZ 159381 Mooloolaba, Australia, Robert Mann. ***Tyrinna*** **Bergh, 1898** *Tyrinna evelinae*, Costa Rica, TMG. ***Noumea*** **Risbec, 1928**: Left *Noumea romeri*, CASIZ 159896, Mooloolaba, Australia Robert Mann. Right *Noumea norba* CASIZ 156661, Philippines, TMG. ***Glossodoris*** **Ehrenberg, 1831** Upper *Glossodoris cincta*, CASIZ 158809, Philippines, Ángel Valdés. Lower *Glossodoris pallida*, CASIZ 157871, Philippines, TMG. ***Goniobranchus*** **Pease, 1866** Upper *Goniobranchus vibratus* CASIZ 175564, Hawaii, USA, Cory Pittman. Middle *Goniobranchus fidelis*, CASIZ 175426, Philippines, Ángel Valdés. Lower Goniobranchus reticualtus CASIZ 156921 TMG. ***‘Doriprismatica’*** **d'Orbigny, 1839** Upper *‘Doriprismatica’ atromarginata*, CASIZ 177237, Philippines, TMG. Lower *‘Doriprismatica’ stellata*, Papua New Guinea, TMG. ***‘Felimida’*** **Marcus, 1971**. Left *Felimida sphoni* CASIZ 175431, Costa Rica, TMG. *Felimida norrisi*, Baja California, TMG. ***Ardeadoris*** **Rudman, 1984**. Upper *Ardeadoris egretta*, CASIZ 157481, TMG. Lower *Ardeadoris angustolutea*, CASIZ 121068, Marshall Islands,Scott Johnson. ***Chromodoris*** **Alder & Hanncock 1855.** Upper *Chromodoris magnifica*, CASIZ 157027, Philippines, TMG. Lower *Chromodoris colemani* CASIZ 158766, Philippines, TMG. ***Diversidoris*** **Rudman, 1987.** Left *Diversidoris crocea*, Philippines, TMG. Right *Diversidoris auriantonodulosa*, New South Wales, Australia, Denis Riek. ***Miamira*** **Bergh, 1874**. Left *Miamira alleni*, CASIZ 180411, Philippines, TMG. Right *Miamira sinuata*, CASIZ 166764, Okinawa, Robert Bolland. ***Ceratosoma*** **Adams & Reeve, 1850.** Left *Ceratosoma* cf. *tenue*, CASIZ 156077, Mooloolaba, Australia, Shireen Fahey. Right *Ceratosoma trilobatum*, CASIZ 173451, Madagascar, TMG. ***Felimare*** **Marcus and Marcus, 1967.** Upper *Felimare bayeri* CASIZ 175461, Bocas del Toro, Panama, Shireen Fahey. Lower *Felimare agassizii*, Baja California, Mexico, TMG. ***Mexichromis*** **Bertsch, 1977.** Upper *Mexichromis antonii*, CASIZ 175436, Costa Rica, TMG. Lower *Mexichromis trilineata*, Philippines, TMG. ***Thorunna*** **Bergh, 1878.** Upper *Thorunna florens*, CASIZ 177094, Vanuatu, Yolanda Camacho. Lower *Thorunna furtiva*, CASIZ 175729, Malaysia, TMG. ***Hypselodoris*** **Stimpson, 1855.** Upper *Hypselodoris zephyra*, Philippines, TMG. Middle *Hypselodoris capensis*, South Africa, TMG. Lower *Hypselodoris obscura*, Jervis Bay, NSW, Australia, Sue Newson.
(JPG)
