# Supplementary material for: Traditional Taxonomic Groupings Mask Evolutionary History: A Molecular Phylogeny and New Classification of the Chromodorid Nudibranchs
Source: PLoS One. 2012 Apr 10;7(4):e33479. doi: 10.1371/journal.pone.0033479 (PMC3323602; doi:10.1371/journal.pone.0033479)
Supplement: Table S1 — Table of Specimens Used in this Study. Specimens used in this study listed by Family. The names in this table reflect current classification not proposed classification (new names are listed in the text). Abbreviations are as follows: CASIZ = California Academy of Sciences, SAM = South Australian Museum, WAM = Western Australian Museum, AM = Australian Museum, ZSM = Zoologische Staatssammlung München, SIO-BIC = Scripps Institute of Oceanography, BioCode = Moorea BioCode Project. (DOCX) [file pone.0033479.s001.docx]

| **Supplementary Table 1. Details for specimens used in this study.** | | | | |
| --- | --- | --- | --- | --- |
| **Specimen** | **Voucher No.** | **Locality** | **COI** | **16s** |
| **Chromodorididae Bergh, 1891** |  |  |  |  |
| ***Ardeadoris egretta*** Rudman, 1984 | CASIZ 157481 | Balayan Bay Batangas Region, Philippines | EU982713 | EU982762 |
| ***Ardeadoris egretta*** Rudman, 1984 | SAM D19257 | Sulawesi, Indonesia | EF535140 | EF34068 |
| *Ardeadoris scottjohnsoni* Bertsch & Gosliner, 1989 | CASIZ 120939 | Pupukea, Oahu Island, Hawaii, USA | EU982714 | EU982763 |
| ***Cadlinella ornatissima*** (Risbec, 1928) | AM C203859 | Heron Island, Great Barrier Reef, Australia | ---- | AY458802 |
| ***Cadlinella ornatissima*** (Risbec, 1928) | CASIZ 175452 | Mooloolaba, Queensland, Australia | EU982728 | EU982779 |
| ***Cadlinella ornatissima*** (Risbec, 1928) | CASIZ 159381 | Mooloolaba, Queensland, Australia | --- | EU982778 |
| *Cadlinella subornatissima* Baba, 1996 | CASIZ 179619 | Marshall Islands | --- | JQ727689 |
| *Ceratosoma alleni* Gosliner, 1996 | CASIZ 180411 | Maricaban Island, Batangas, Philippines | --- | JQ727690 |
| *Ceratosoma amoenum* (Cheeseman, 1886) | CASIZ 121274 | Rottnest, Island, Western Australia | EU982729 | EU982780 |
| *Ceratosoma gracillimum* Semper in Bergh, 1876 | CASIZ 157206 | Caban Island, Batangas Region, Philippines | JQ727822 | JQ727691 |
| *Ceratosoma ingozi*  Gosliner, 1996 | CASIZ 176194 | False Bay, Western Cape Province, South Africa | --- | JQ727692 |
| *Ceratosoma ingozi*  Gosliner, 1996 | CASIZ 176195 | False Bay, Western Cape Province, South Africa | JQ727823 | JQ727693 |
| *Ceratosoma magnificum* (Eliot, 1910) | CASIZ 169951 | Mooloolaba, Queensland, Australia | EU982731 | EU982781 |
| *Ceratosoma magnificum* (Eliot, 1910) | CASIZ 168866 | Mooloolaba, Queensland, Australia | --- | JQ727694 |
| *Ceratosoma miamiranum* (Bergh, 1875) | CASIZ 173407 | Nosy Kalakjoro, Iles de Radama, Madagascar | EU982732 | EU982782 |
| *Ceratosoma sinuatum* (van Hasslet, 1824) | CASIZ 069927 | Onna Village, Ryukyu Island, Okinawa, Japan | EU982733 | EU982783 |
| *Ceratosoma sinuatum* (van Hasslet, 1824) | CASIZ 166764 | Napili Bay, Maui, Hawaii | --- | JQ727695 |
| *Ceratosoma tenue* Abraham, 1876 | CASIZ 163772 | Makena, Maui, Hawaii, USA | --- | JQ727696 |
| *Ceratosoma cf. tenue* Abraham, 1876 | CASIZ 156077 | Mooloolaba, Queensland, Australia | JQ727824 | JQ727697 |
| ***Ceratosoma trilobatum*** J.E.Gray, 1850 | SAM D19259 | Amity, Queensland, Australia | EF535142 | EF534070 |
| ***Ceratosoma trilobatum*** J.E. Gray, 1850 | CASIZ 173451 | Nosi Valiha, Iles de Radama, Madagascar | EU982730 | EU982784 |
| *Ceratosoma* sp.2 *in* Gosliner, Behrens & Valdés, 2009 | CASIZ 173416 | Nosy Kalakajoro, Iles de Radama, Madagascar | JQ727825 | JQ727698 |
| Chromodorididae | BIOCODE2937 | Moorea, French Polynesia | --- | --- |
| *Chromodoris africana* Eliot, 1904 | CASIZ 173653 | Nosi Valiha, Iles de Radama, Madagascar | JQ727826 | JQ727699 |
| *Chromodoris albopunctata* (Garrett, 1879) | CASIZ 121268 | Rottnest Island, Western Australia, Australia | JQ727827 | JQ727700 |
| *Chromodoris albopustulosa* (Pease, 1860) | CASIZ 142953 | Mala Wharf, Maui, Hawaii | JQ727828 | JQ727701 |
| *Chromodoris* cf*. alderi* Collngwood, 1881 | WAM 121272 | Western Australia | --- | JQ727702 |
| *Chromodoris alternata* (Burn, 1957) | SAM D19281 | Port Phillip Bay, Victoria, Australia | EF535120 | AY458800 |
| *Chromodoris ambiguus* (Rudman, 1987) | SAM D19260 | Port Phillip Bay, Victoria, Australia | EF535119 | AY458801 |
| *Chromodoris annae* Bergh, 1877 | CASIZ 121261 | Rottnest Island, Western Australia, Australia | JQ727830 | JQ727703 |
| *Chromodoris annae* Bergh, 1877 | CASIZ 158677 | Caban Islands, Batangas, Philippines | JQ727829 | JQ727704 |
| *Chromodoris aspersa* (Gould, 1852) | CASIZ 174975 | Napili Bay, Maui, Hawaii | --- | JQ727705 |
| *Chromodoris aspersa* (Gould, 1852) | SAM D19282 | Mooloolaba, Queensland, Australia | --- | AY458813s |
| *Chromodoris boucheti* Rudman, 1982 | CASIZ 173594 | Iles de Radama, Madagascar | JQ727831 | JQ727706 |
| **Specimen** | **Voucher No.** | **Locality** | **COI** | **16s** |
| *Chromodoris burni* Rudman, 1982 | CASIZ 179255 | Espiritu Santo Island, Vanuatu | --- | JQ727707 |
| *Chromodoris clenchi* (Russell, 1935) | CASIZ 175544 | Salt Creek, Bocas del Toro, Panama | JQ727832 | JQ727708 |
| *Chromodoris coi* (Risbec, 1956) | CASIZ 158683 | Eagle Point, Batangas, Philippines | EU982734 | EU982785 |
| *Chromodoris colemani* Rudman, 1982 | CASIZ 158766 | Arthur’s Point, Batangas, Philippines | JQ727833 | JQ727709 |
| *Chromodoris collingwoodi* Rudman, 1987 | CASIZ 139597 | Tulamben, Bali, Indonesia | JQ727834 | JQ727710 |
| *Chromodoris collingwoodi* Rudman, 1987 | SAM D19284 | Moreton Bay, Queensland, Australia | --- | AY731181 |
| *Chromodoris cf. collingwoodi* Rudman, 1987 | CASIZ 159382 | Mooloolaba, Queensland, Australia | JQ727835 | JQ727711 |
| *Chromodoris daphne* (Angus, 1864) | SAM D19284 | Moreton Bay, Queensland, Australia | --- | AY458804 |
| *Chromodoris decora* (Pease, 1860) | CASIZ 157025 | Caban Island, Batangas, Philippines | EU982735 | EU982786 |
| *Chromodoris dianae* Gosliner & Behrens, 1988 | CASIZ 158686 | Eagle Point, Batangas, Philippines | JQ727836 | JQ727712 |
| *Chromodoris elisabethina* Bergh, 1877 | CASIZ 144032a | Mooloolaba, Queensland, Australia | JQ727837 | JQ727713 |
| *Chromodoris epicurea* (Basedow & Hedley, 1905) | SAM D19285 | Tasmania, Australia | EF535114 | AY458804 |
| *Chromodoris fidelis* (Kelaart, 1858) | CASIZ 175556 | Nosy Kalakajoro, Iles de Radama, Madagascar | JQ727839 | JQ727714 |
| *Chromodoris fidelis* (Kelaart, 1858) | CASIZ 175426 | Eagle Point, Batangas, Philippines | JQ727838 | JQ727715 |
| *Chromodoris geminus* Rudman, 1987 | CASIZ 173434 | Nosi Faly, Iles de Radama, Madagascar | JQ727840 | JQ727716 |
| *Chromodoris geometrica* Risbec, 1928 | CASIZ 175549 | Nosi Valiha, Iles de Radama, Madagascar | JQ727842 | JQ727717 |
| *Chromoodoris geometrica* Risbec, 1928 | CASIZ 144023 | Mooloolaba, Queensland, Australia | JQ727841 | JQ727718 |
| *Chromoodoris geometrica* Risbec, 1928 | SAM D19286 | Mooloolaba, Queensland, Australia | --- | AY458805 |
| *Chromodoris hamiltoni* Rudman, 1977 | CASIZ 173408 | Nosi Valiha, Iles de Radama, Madagascar | JQ727843 | JQ727719 |
| *Chromodoris heatherae*  Gosliner, 1994 | CASIZ 175546 | Cape Peninsula, South Africa | JQ727844 | JQ727720 |
| *Chromodoris hinatuaensis* Gosliner & Behrens, 1998 | CASIZ 158346 | Balayan Bay, Batangas, Philippines | JQ727845 | JQ727721 |
| *Chromodoris joshi* Gosliner & Behrens, 1998 | CASIZ 156943 | Caban Island, Batangas, Philippines | JQ727846 | JQ727722 |
| *Chromodoris krohni* (Verany, 1846) | --- | Spain, North Atlantic | AF249805 | AF249239 |
| *Chromodoris kuiteri* Rudman, 1982 | CASIZ 144025 | Mooloolaba, Queensland, Australia | --- | JQ727723 |
| *Chromodoris kuiteri* Rudman, 1982 | SAM D19287 | Mooloolaba, Queensland, Australia | AF249804 | --- |
| *Chromodoris kuiteri* Rudman, 1982 | --- | Great Barrier Reef, Australia | --- | AF2499240 |
| *Chromodoris kuniei* Pruvot-Fol, 1930 | CASIZ 157485 | Balayan Bay, Batangas, Philippines | --- | JQ727724 |
| *Chromodoris kuniei* Pruvot-Fol, 1930 | CASIZ 159383 | Mooloolaba, Queensland, Australia | --- | JQ727725 |
| *Chromodoris kuniei* Pruvot-Fol, 1930 | SAM D19261 | Heron Island, Great Barrier Reef, Australia | EF535112 | AY458807 |
| *Chromodoris leopardus* Rudman, 1987 | CASIZ 159384 | Mooloolaba, Queensland, Australia | JQ727847 | JQ727726 |
| *Chromodoris leopardus* Rudman, 1987 | SAM D19288 | Mooloolaba, Queensland, Australia | EF535116 | AY458808 |
| *Chromodoris lochi* Rudman, 1982 | CASIZ 158684 | Eagle Pt., Batangas, Philippines | JQ727848 | JQ727727 |
| *Chromodoris lochi* Rudman, 1982 | CASIZ 167968 | D’Entrecasteaux Islands, Papua New Guinea | JQ727850 | JQ727728 |
| *Chromodoris lochi* Rudman, 1982 | CASIZ 167973 | Aniwa Island, Vanuatu | JQ727851 | JQ727729 |
| *Chromodoris lochi* Rudman, 1982 | Paris Museum | Lifou, New Caledonia | JQ727849 | JQ727730 |
| *Chromodoris lochi* Rudman, 1982 | SAM 19289 | Mooloolaba, Queensland, Australia | --- | AY458810 |
| **Specimen** | **Voucher No.** | **Locality** | **COI** | **16s** |
| *Chromodoris luteorosea* (von Rapp, 1827) | --- | Cadiz, Andalusia, Spain | AJ223259 | AJ225183 |
| ***Chromodoris magnifica*** (Quoy & Gaimard, 1832) | CASIZ 144119 | Mooloolaba, Queensland, Australia | JQ727852 | JQ727731 |
| ***Chromodoris magnifica*** (Quoy & Gaimard, 1832) | SAM D19290 | Whitsundays, Queensland, Australia | EF535110 | EF534042 |
| ***Chromodoris magnifica*** (Quoy & Gaimard, 1832) | CASIZ 157027 | Maricaban Island, Batangas, Philippines | EU982736 | EU982787 |
| *Chromodoris norrisi* Farmer, 1963 | ---- | Baja California, Mexico | EU982737 | EU982788 |
| *Chromodoris preciosa* (Kelaart, 1858) | CASIZ 176752 | Pulau Tioman, Pulau Gut, Malaysia | --- | JQ727732 |
| *Chromodoris purpurea* (Risso in Guérin, 1831) | ---- | Cadiz, Andalusia, Spain | AF249815 | AJ225183 |
| *Chromodoris quadricolor* (Ruppell & Leuckhart, 1828) | ---- | Red Sea, Eygpt | --- | AF249241 |
| *Chromodoris reticulata* (Quoy & Gaimard, 1832) | CASIZ 156921 | Maricaban Island, Batangas, Philippines | JQ727853 | JQ727733 |
| *Chromodoris roboi* Gosliner & Behrens, 1998 | --- | Heron Island, Queensland, Australia | --- | AY458814 |
| *Chromodoris cf. roboi* Gosliner & Behrens, 1998 | CASIZ 121275 | Rottnest Island, Western Australia, Australia | JQ727854 | JQ727734 |
| *Chromodoris sinensis*  Rudman, 1985 | CASIZ 175727 | Pulau Tioman, Pulau Gut, Malaysia | --- | JQ727735 |
| *Chromodoris sphoni* (Marcus, Ev., 1971) | CASIZ 175431 | Punta Carbon, Guanacaste, Costa Rica | --- | JQ727736 |
| *Chromodoris splendida* (Angus, 1864) | CASIZ 146039 | Mooloolaba, Queensland, Australia | EU982738 | EU982789 |
| *Chromodoris splendida* (Angus, 1864) | SAM D19292 | Mooloolaba, Queensland, Australia | EF535115 | AY458815 |
| *Chromodoris striatella* Bergh, 1876 | CASIZ 121288 | Rottnest Island, Western Australia, Australia | JQ727855 | JQ727737 |
| *Chromosdoris striatella* Bergh, 1876 | SAM D19293 | Mooloolaba, Queensland, Australia | EF535111 | AY458809 |
| *Chromodoris strigata* Rudman, 1982 | CASIZ 158260 | Maricaban Island, Batangas, Philippines | JQ727856 | JQ727738 |
| *Chromodoris strigata* Rudman, 1982 | CASIZ 175558 | Nosi Kalakjoro, Iles de Radama, Madagascar | JQ727857 | JQ727739 |
| *Chromodoris strigata* Rudman, 1982 | SAM 19294 | Heron Island, Great Barrier Reef, Australia | --- | AY458816 |
| *Chromodoris tasmaniensis* Bergh, 1905 | SAM 19295 | Triabunna, Tasmania, Australia | EF535113 | AY458817 |
| *Chromodoris verrieri* (Crosse, 1875) | CASIZ 158796 | Maricaban Island, Batangas, Philippines | JQ727858 | JQ727740 |
| *Chromodoris vibrata* (Pease, 1860) | CASIZ 175564 | Maui, Hawaii, USA | JQ727859 | JQ727741 |
| *Chromodoris westraliensis* (O'Donoghue, 1924) | CASIZ 121263 | Rottnest Island, Western Australia, Australia | JQ727860 | JQ727742 |
| *Chromodoris willani* Rudman, 1982 | CASIZ 159385 | Mooloolaba, Queensland, Australia | JQ727861 | JQ727743 |
| *Chromodoris willani* Rudman, 1982 | ---- | Mooloolaba, Queensland, Australia | JQ727862 | JQ727744 |
| ***Digidentis cf.arbutus*** (Burn, 1961) | ---- | Point Puer, Tasmania, Australia | EF535143 | EF534043 |
| *Digidentis kulonba* (Burn, 1966) | CASIZ 121285 | Western Australia | JQ727863 | JQ727745 |
| *Digidentis perplexa* (Burn, 1957) | SIO-BIC M11633 | Bicheno, Tasmania, Australia | EF535144 | EF534044 |
| ***Diversidoris aurantinodulosa*** Rudman, 1987 | SAM D19263 | Mooloolaba, Queensland, Australia | EF535141 | EF534069 |
| ***Durvilledoris lemniscata*** (Quoy & Gaimard, 1832) | CASIZ 178377 | Belmoul lagoon, Espiritu Santo Island, Vanuatu | ---- | EU982790 |
| *Durvilledoris pusilla* (Bergh, 1874) | ---- | Tab Island, Papua New Guinea | AJ223269 | AJ225193 |
| *Durvilledoris pusilla* (Bergh, 1874) | CASIZ 158807 | Calumpaun Peninsula, Batangas, Philippines | EU982739 | EU982791 |
| *Durvilledoris similaris* Rudman, 1986 | --- | Lizard Island, Queensland, Australia | EF535128 | EF534055 |
| *Glossodoris atromarginata* (Cuvier, 1804) | ---- | Great Barrier Reef, Australia | AF249789 | --- |
| *Glossodoris atromarginata* (Cuvier, 1804) | CASIZ 177237 | Caban Island, Batangas, Philippines | JQ727864 | JQ727746 |
| **Specimen** | **Voucher No.** | **Locality** | **COI** | **16s** |
| *Glossodoris averni* (Rudman, 1985) | CASIZ 159389 | Mooloolaba, Queensland, Australia | JQ727865 | JQ727747 |
| *Glossodoris baumanni* (Bertsch, 1970) | CASIZ 175433 | Playa Tamarindo, Costa Rica | JQ727866 | JQ727748 |
| *Glossodoris cincta* (Bergh, 1888) | ---- | Heron Island, Queensland, Australia | EF535136 | EF534064 |
| *Glossodoris cincta* (Bergh, 1888) | CASIZ 158809 | Calumpan Peninsula, Batangas, Philippines | EU982740 | EU982792 |
| *Glossodoris cincta* (Bergh, 1888) | CASIZ 173394 | Nosi Valiha, Iles de Radama, Madagascar | JQ727867 | JQ727749 |
| *Glossodoris*  cf. *cincta* (Bergh, 1888) | CASIZ 173433 | Nosi Valiha, Iles de Radama, Madagascar | JQ727868 | JQ727750 |
| *Glossodoris dalli* (Bergh, 1879) | CASIZ 175428 | Punta Carbon, Guanacaste, Costa Rica | EU982741 | EU982793 |
| *Glossodoris dalli* (Bergh, 1879) | CASIZ 175439 | Islas Tres Hermanas, Costa Rica | JQ727869 | JQ727751 |
| *Glossodoris edmundsi*  Cervera et al, 1989 | CASIZ 179385 | Pedra Adalio, Principe Island, Sao Tome and Principe | HM162686 | HM162595 |
| *Glossodoris edmundsi* Cervera et al, 1989 | --- | Ilheu Cabra, Sao Tome Island, Sao Tome and Principe | EF535133 | EF534064 |
| *Glossodoris hikuerensis* (Pruvot-Fol, 1954) | CASIZ 158341 | Caban Island, Batangas, Philippines | JQ727870 | JQ727752 |
| ***Glossodoris pallida*** (Ruppell & Leuckhart, 1828) | SIO-BIC 11635 | Heron Island, Queensland, Australia | EF535138 | EF534066 |
| ***Glossodoris pallida*** (Ruppell & Leuckhart, 1828) | CASIZ 175548 | Kalakajoro, Iles de Radama, Madagascar | EU982742 | EU982794 |
| ***Glossodoris pallida*** (Ruppell & Leuckhart, 1828) | CASIZ 175554 | Nosi Faly, Iles de Radama, Madagascar | JQ727872 | JQ727753 |
| ***Glossodoris pallida*** (Ruppell & Leuckhart, 1828) | CASIZ 157871 | Balayan Bay, Batangas, Philippines | JQ727871 | JQ727754 |
| *Glossodoris pullata* Rudman, 1995 | SIO-BIC MII634 | Heron Island, Queensland, Australia | EF535137 | EF534065.2 |
| *Glossodoris rubroannulata* Rudman, 1986 | CASIZ 142785 | Heron Island, Queensland, Australia | JQ727873 | JQ727755 |
| *Glossodors rufomarginata* (Bergh, 1890) | CASIZ 175565 | Black Rock, Maui, Hawaii | JQ727874 | JQ727756 |
| *Glossodors rufomarginata* (Bergh, 1890) | Paris Museum | Lifou, New Caledonia | JQ727875 | --- |
| *Glossodors rufomarginata* (Bergh, 1890) | CASIZ 121284 | Rottnest Island, Western Australia, Australia | JQ727876 | JQ727757 |
| *Glossodoris sedna* (Marcus & Marcus, 1967) | SIO-BIO M11636 | Florida Keys, Florida, USA | EF535134 | EF534062 |
| *Glossodoris sedna* (Marcus & Marcus, 1967) | CASIZ 175430 | Punta Carbon, Costa Rica | JQ727877 | JQ727758 |
| *Glossodoris sedna* (Marcus & Marcus, 1967) | CASIZ 175435 | Playa Real, Costa Rica | JQ727879 | JQ727759 |
| *Glossodoris sedna* (Marcus & Marcus, 1967) | CASIZ 175446 | Isla Ulva, Golfo de Chiriqui, Pacific Coast, Panama | JQ727878 | JQ727760 |
| *Glossodoris sibogae* (Bergh, 1905) | BioCode 2687 | Moorea French Polynesia | --- |  |
| *Glossodoris sibogae* (Bergh, 1905) | SIO-BIO M11637 | French Polynesia | EF535135 | EF534063 |
| *Glossodoris stellatus* Rudman, 1986 | CASIZ 167963 | Nabwageta Island, Papua New Guinea | JQ727880 | JQ727761 |
| *Glossodoris tomsmithi* Bertsch & Gosliner, 1989 | CASIZ 175566 | Black Rock, Maui, Hawaii, USA | JQ727881 | JQ727762 |
| *Glossodoris undaurum* Rudman, 1985 | CASIZ 121262 | Dampier, Western Australia, Australia | JQ727882 | JQ727763 |
| *Hypselodoris agassizii* (Bergh, 1894) | CASIZ 175441 | Baja California, Mexico | JQ727883 | JQ727764 |
| *Hypselodoris agassizii* (Bergh, 1894) | CASIZ 175429 | Punta Carbon, Coast Rica | JQ727884 | JQ727765 |
| *Hypselodoris apolegma* (Yonow, 2001) | CASIZ 070042 | Onna Village, Ryukyu Islands, Okinawa, Japan | JQ727886 | JQ727766 |
| *Hypselodoris apolegma* (Yonow, 2001) | CASIZ 157479 | Maricaban Island, Batangas, Philippines | JQ727885 | JQ727767 |
| *Hypselodoris bayeri*  (Marcus & Marcus, 1967) | CASIZ 175461 | Salt Creek, Bocas del Toro, Panama | --- | JQ727768 |
| *Hypselodoris bennetti* (Angus, 1864) | --- | Wilson’s Promatory, Victoria, Australia | EF535131 | EF534059 |
| *Hypselodoris bertschi* Gosliner & Johnson, 1999 | CASIZ 181328 | Mala Wharf, Maui , Hawaii, USA | --- | JQ727769 |
| **Specimen** | **Voucher No.** | **Locality** | **COI** | **16s** |
| *Hypselodoris bilineata* (Pruvot-Fol, 1953) | --- | Madiera, Portugal | EF535125 | EF534052 |
| *Hypselodoris bollandi* Gosliner & Johnson, 1999 | CASIZ 079211 | Maekizaki, Ryukyu Island, Okinawa, Japan | --- | JQ727770 |
| *Hypselodoris bollandi* Gosliner & Johnson, 1999 | CASIZ 157027 | Maricaban Island, Batangas, Philippines | JQ727887 | JQ727771 |
| *Hypselodoris bullocki* (Collingwood, 1881) | CASIZ 156932 | Maricaban Island, Batangas, Philippines | EU982743 | EU982795 |
| *Hypselodoris bullocki* (Collingwood, 1881) | CASIZ 069997 | Onna Village, Ryukyu Islands, Okinawa, Japan | JQ727888 | JQ727772 |
| *Hypselodoris californiensis* (Bergh, 1879) | CASIZ 175442 | Baja California, Mexico | EU982744 | EU982796 |
| *Hypselodoris capensis* (Barnard, 1927) | CASIZ 176393 | Eastern Cape Province, South Africa | --- | JQ727773 |
| *Hypselodoris elegans picta* (Schultz, 1836) | --- | North Atlantic, Spain | AF249787 | AF249238 |
| *Hypselodoris emma*  Rudman, 1977 | CASIZ 156654 | Dead Point, Batangas, Philippines | JQ727889 | JQ727774 |
| *Hypselodoris emma*  Rudman, 1977 | CASIZ 175552 | Iles de Radama, Madagascar | JQ727890 | JQ727775 |
| *Hypselodoris infucata* (Ruppell & Leuckhart, 1828) | CASIZ 175551 | Iles de Radama, Madagascar | JQ727891 | JQ727776 |
| *Hypselodoris jacksoni* Wilson & Willan, 2007 | CASIZ 146036 | Mooloolaba, Queensland, Australia | JQ727893 | JQ727777 |
| *Hypselodoris jacksoni* Wilson & Willan, 2007 | CASIZ 175450 | Mooloolaba, Queensland, Australia | JQ727892 | JQ727778 |
| *Hypselodoris kaname*  Baba, 1994 | CASIZ 107221 | Seragaki Tombs, Ryukyu Islands, Okinawa, Japan | --- | JQ727779 |
| *Hypselodoris kaname*  Baba, 1994 | CASIZ 177776 | Maricaban Island, Batangas, Philippines | --- | JQ727780 |
| *Hypselodoris krakatoa* Gosliner & Johnson, 1999 | CASIZ 177371 | Mora Mora Village, Madagascar | --- | JQ727781 |
| *Hypselodoris krakatoa* Gosliner & Johnson, 1999 | CASIZ 175726 | Rayner’s Rock, Pulau Aur, Malaysia | --- | JQ727782 |
| *Hypselodoris krakatoa* Gosliner & Johnson, 1999 | CASIZ 178350 | Long Dong, Taipei County, Taiwan | --- | JQ727783 |
| *Hypselodoris maculosa* (Pease, 1871) | CASIZ 139595 | Tulamben, Bali, Indonesia | JQ727894 | JQ727784 |
| *Hypselodoris maculosa* (Pease, 1871) | CASIZ 175550 | Nosi Valiha, Iles de Radama, Madagascar | JQ727895 | JQ727785 |
| *Hypselodoris*  cf. *maculosa* sp.1 Gosliner et al, 2009 | Photo voucher | Iles de Radama, Madagascar | JQ727896 | JQ727786 |
| *Hypselodoris marci* (Marcus, 1970) | CASIZ 175545 | Crawl Cay, Bocas del Toro, Panama | ---- | JQ727787 |
| *Hypselodoris maritima* (Baba, 1949) | CASIZ 175449 | Mooloolaba, Queensland, Australia | JQ727897 | JQ727788 |
| *Hypselodoris midatlantica* Gosliner, 1990 | CASIZ 175443 | Islotes do Martinha, Algarve, Portugal | JQ727898 | JQ727789 |
| *Hypselodoris cf. nigrolineata*(Eliot, 1904) | WAM 12622 | Dampier, Western Australia, Australia | JQ727899 | JQ727790 |
| ***Hypselodoris obscura*** Stimpson, 1855 | AM C379393 | Amity, Queensland, Australia | ---- | EF534058 |
| ***Hypselodoris obscura*** Stimpson, 1855 | SIO-BIC M11638 | Amity, Queensland, Australia | EF535130 | ---- |
| ***Hypselodoris obscura*** Stimpson, 1855 | CASIZ 144029 | Mooloolaba, Queensland, Australia | EU982745 | EU982797 |
| *Hypselodoris orsinii* (Verany, 1846) | --- | Cadiz, Adalusia, Spain | AJ223265 | AJ225189 |
| *Hypselodoris paulinae* Gosliner & Johnson, 1999 | CASIZ 167984 | Molokini, Maui, Hawaii, USA | EU982746 | EU982798 |
| *Hypselodoris picta verdensis* Ortea, et al, 1996 | CASIZ 179384 | Pedra Adalio, Principe Island, Sao Tome and Principe | HM162685 | HM162594 |
| *Hypselodoris purpureomaculosa*  Hamatani, 1995 | CASIZ 158675 | Caban Island, Batangas, Philippines | JQ727900 | JQ727791 |
| *Hypselodoris reidi* Gosliner & Johnson, 1999 | CASIZ 157143 | Balayan Bay, Batangas, Philippines | JQ727901 | JQ727792 |
| *Hypselodoris ruthae* Marcus & Hughes, 1974 | CASIZ 175458 | Hospital Point, Bocas del Toro, Panama | EU982747 | EU982799 |
| *Hypselodoris villafranca* (Risso, 1818) | --- | Cadiz, Andalusia, Spain | AJ223266 | AJ225190 |
| *Hypselodoris villafranca* (Risso, 1818) | CASIZ 185127 | Algarve, Portugal | --- | JQ727793 |
| **Specimen** | **Voucher No.** | **Locality** | **COI** | **16s** |
| *Hypselodoris whitei* (Adams & Reeve, 1850) | CASIZ 159391 | Mooloolaba, Queensland, Australia | JQ727903 | JQ727794 |
| *Hypselodoris whitei* (Adams & Reeve, 1850) | CASIZ 173442 | Madagascar | JQ727902 | JQ727795 |
| *Hypselodoris zephyra* Gosliner & Johnson, 1999 | CASIZ 139599 | Tulamben, Bali, Indonesia | JQ727904 | JQ727796 |
| *Hypselodoris zephyra* Gosliner & Johnson, 1999 | CASIZ 175555 | Nosy Faly, Iles de Radama, Madagascar | JQ727905 | JQ727797 |
| *Hypselodoris zephyra* Gosliner & Johnson, 1999 | --- | Mooloolaba, Queensland, Australia | EF535129 | EF534057 |
| *Hypselodoris zephyra* Gosliner & Johnson, 1999 | SIO-BIC M11639 | Cook Island, New South Wales, Australia |  | EF534056 |
| ***Mexichromis antonii***  (Bertsch, 1976) | CASIZ 175432 | Playa Real, Costa Rica | EU982748 | EU982800 |
| ***Mexichromis antonii***  (Bertsch, 1976) | CASIZ 175436 | Playa Tamarindo, Costa Rica |  | JQ727798 |
| *Mexichromis festiva* (Angus, 1864) | SIO-BIC M11640 | Coffs Harbour, New South Wales, Australia | EF535124 | EF534051 |
| *Mexichromis kempfi* (Ev. Marcus, 1970) | CASIZ 185129a | Los Huecos, Punta Uva, Costa Rica | ---- | JQ727799 |
| *Mexichromis kempfi* (Ev. Marcus, 1970) | CASIZ 185129b | Los Huecos, Punta Uva, Costa Rica | ---- | JQ727800 |
| *Mexichromis kempfi* (Ev. Marcus, 1970) | SIO-BIC M11641 | Florida Keys, Florida, USA | EF535121 | EF34047 |
| *Mexichromis macropus* Rudman, 1983 | WAM 12634 | Dampier, Western Australia | EF535123 | EF534050 |
| *Mexichromis macropus* Rudman, 1983 | --- | Dampier, Western Australia | --- | JQ727801 |
| *Mexichromis mariei* (Crosse, 1872) | Photo voucher | Twin Rocks, Batangas, Philippines | EU982749 | EU982801 |
| *Mexichromis mariei* (Crosse, 1872) | SAM D19268 | Amity, Queensland, Australia | --- | EF534049 |
| *Mexichromis multituberculata* (Baba, 1953) | Photo voucher | Twin Rocks, Batangas, Philippines | JQ727907 | --- |
| *Mexichromis multituberculata* (Baba, 1953) | CASIZ 139601 | Tulamben, Bali, Indonesia | JQ727906 | JQ727802 |
| *Mexichromis porterae* (Cockerell, 1902) | SIO-BIC M11642 | Palos Verdes, California, USA | EF535139 | EF534067 |
| *Noumea alboannulata* Rudman, 1986 | CASIZ 177378 | Maricaban Island, Batangas, Philippines | ---- | JQ727803 |
| *Noumea angustolutea* Rudman, 1990 | CASIZ 121068 | Kwajalein Atoll, Marshall Islands | ---- | JQ727804 |
| *Noumea crocea* Rudman, 1986 | Photo voucher | Twin Rocks, Batangas, Philippines | EU982750 | EU982802 |
| *Noumea flava* (Eliot, 1904) | CASIZ 142951 | Mala Wharf, Maui, Hawaii, USA | --- | JQ727805 |
| *Noumea flava* (Eliot, 1904) | CASIZ 157150 | Balayan Bay, Batangas, Philippines | --- | JQ727806 |
| *Noumea haliclona* (Burn, 1957) | SAM D19269 | Australia: Port Phillip Bay, VIC | EF535117 | EF534045 |
| *Noumea laboutei* Rudman, 1986 | CASIZ 176851 | Asuleka Island, Espiritu Santo Island, Vanuatu | --- | JQ727807 |
| *Noumea norba* Marcus & Marcus, 1970 | CASIZ 069976 | Onna Village, Ryukyu Islands, Okinawa, Japan | --- | JQ727808 |
| *Noumea protea* Marcus & Marcus, 1970 | CASIZ 176242 | Gordon's Bay, False Bay, South Africa | --- | JQ727809 |
| *Noumea purpurea* Baba, 1949 | CASIZ 070063 | Onna Village, Ryukyu Islands, Okinawa, Japan | --- | JQ727810 |
| ***Noumea romeri*** Risbec, 1928 | CASIZ 159896 | Mooloolaba, Australia | EU982751 | EU982803 |
| *Noumea simplex* (Pease, 1871) | CASIZ 175553 | Nosi Faly, Iles de Radama, Madagascar | EU982752 | EU982804 |
| *Noumea varians* (Pease, 1871) | CASIZ 156661 | Dead Point, Batangas, Philippines | JQ727909 | JQ727812 |
| *Noumea varians* (Pease, 1871) | CASIZ 175560 | Maalaea, Maui, Hawaii, USA | JQ727908 | JQ727811 |
| *Pectenodoris aurora* Johnson & Gosliner, 1998 | CASIZ 175732 | Tiger Point, South China Sea, Malyasia | ---- | EU982805 |
| ***Pectenodoris trilineata*** (Adams & Reeve, 1850) | Photo voucher | Bethlehem, Batangas, Philippines | EU982753 | EU982806 |
| ***Pectenodoris trilineata*** (Adams & Reeve, 1850) | ----- | Heron Island, Queensland, Australia | EF535122 | EF534048 |
| **Specimen** | **Voucher No.** | **Locality** | **COI** | **16s** |
| *Risbecia imperialis* (Pease, 1860) | CASIZ 142952 | Mala Wharf, Maui, Hawaii, USA | EU982754 | EU982807 |
| *Risbecia imperialis* (Pease, 1860) | CASIZ 175561 | Mala wharf, Maui, Hawaii, USA | JQ727910 | JQ727813 |
| *Risbecia imperialis* (Pease, 1860) | CASIZ175562 | Maui, Hawaii, USA | JQ727911 | JQ727814 |
| *Risbecia pulchella* Rüppell & Leuckart, 1828 | CASIZ 104979 | Aliwal Shoals, Natal, South Africa | --- | JQ727815 |
| ***Risbecia tyoni*** (Garrett, 1873) | Paris Museum | Lifou, New Caledonia | EU982755 | EU982808 |
| ***Risbecia tryoni*** (Garrett, 1873) | SIO-BIC M11643 | Heron Island, Queensland, Australia | ---- | EF534060 |
| ***Risbecia tryoni*** (Garrett, 1873) | ----- | Sulawesi, Indonesia | EF535132 | ---- |
| *Thorunna australis* (Risbec,, 1928) | CASIZ 069910 | Onna Village, Ryukyu Islands, Okinawa, Japan | JQ727912 | JQ727816 |
| *Thorunna daniellae* (Kay & Young, 1969) | CASIZ 170055 | Maalaea, Maui, Hawaii, USA | EU982756 | EU982809 |
| *Thorunna florens* (Baba, 1949) | CASIZ 158258 | Maricaban Island, Batangas, Philippines | JQ727913 | --- |
| *Thorunna florens* (Baba, 1949) | CASIZ 177094 | Espiritu Santo Island, Vanuatu | --- | JQ727817 |
| ***Thorunna furtiva*** Bergh, 1878 | CASIZ 175729 | Pineapple Point, Malaysia | ---- | EU982810 |
| ***Thorunna furtiva*** Bergh, 1878 | ---- | Sulawesi, Indonesia | EF535126 | EF534053 |
| *Thorunna halourga* Johnson & Gosliner, 2001 | CASIZ 177252 | Maricaban Island, Batangas, Philippines | --- | JQ727818 |
| *Thorunna montrouzieri* Rudman, 1995 | CASIZ 181466 | Kwajalein Atoll, Marshall Islands | --- | JQ727819 |
| *Thorunna punicea* Rudman, 1995 | CASIZ 173597 | Iles de Radama, Madagascar | JQ727914 | JQ727820 |
| *Thorunna purpureopedis* Rudman & Johnson, 1985 | CASIZ 185128 | Kwajalein Atoll, Marshall Islands | --- | JQ727821 |
| *Tyrinna evelinae* (Marcus, 1958) | CASIZ 175440 | Playa Ventana, Costa Rica | EU982757 | EU982811 |
| *Tyrinna evelinae* (Marcus, 1958) | --- | Costa Rica | EU982758 | EU982812 |
| ***Tyrinna nobilis*** Bergh, 1898 | ZSM-M20050508 | Region de Los Lagos, Chile | EF535127 | EF534054 |
| ***Verconia verconis*** (Basedow & Hedley, 1905) | SAM D19270 | Port Philip Bay, Victoria, Australia | EF535118 | EF534046 |
| Actinocyclidae O’Donoghue 1929 |  |  |  |  |
| *Actinocyclus verrucosus* Ehrenberg, 1831 | SAM D19274 | Mooloolaba, Queensland, Australia | ---- | AY458799 |
| *Hallaxa indecora* (Bergh, 1905) | SAM D19275 | Mooloolaba, Queensland, Australia | ---- | EF534071 |
| *Hallaxa iju* Gosliner & Johnson, 1994 | CASIZ 175559 | Five graves, Maui, Hawaii | EU982759 | EU982813 |
| *Hallaxa translucens* Gosliner & Johnson, 1994 | CASIZ 173447 | Nosi Valiha, Iles de Radama, Madagascar | EU982760 | EU982814 |
| Discodorididae Bergh 1891 |  |  |  |  |
| *Peltodoris atromaculata*  Bergh, 1880 | ---- | ----- | AF120637 | DQ280054 |
| *Peltodoris nobilis*  (MacFarland, 1905) | CASIZ 182223 | Pillar Point, San Mateo County, California, USA | EU982761 | EU982816 |
| *Platydoris argo* (Linnaeus, 1767) | ---- | Ceuta, Straight of Gilbraltar, Spain | AY345037 | AY345037 |
| Doridiade Raffinesque 1815 |  |  |  |  |
| *Doris kerguelensis* (Bergh, 1894) | ---- | Weddell Sea, Antarctica | AF249780 | AF249233 |

Specimens used in this study listed by Family. The names in this table reflect current classification not proposed classification (new names are listed in the text and Supplementary Table 2). Abbreviations are as follows: CASIZ=California Academy of Sciences, SAM=South Australian Museum, WAM= Western Australian Museum, AM= Australian Museum, ZSM= Zoologische Staatssammlung München, SIO-BIC= Scripps Institute of Oceanography, BioCode= Moorea BioCode Project
