# Supplementary material for: Traditional Taxonomic Groupings Mask Evolutionary History: A Molecular Phylogeny and New Classification of the Chromodorid Nudibranchs
Source: PLoS One. 2012 Apr 10;7(4):e33479. doi: 10.1371/journal.pone.0033479 (PMC3323602; doi:10.1371/journal.pone.0033479)
Supplement: Table S2 — New Classification of the Chromodorididae with synonyms. Generic names and type species in bold and the most recent genus membership follows. Listing order follows phylogeny. (DOCX) [file pone.0033479.s002.docx]

| **Name** | **Authority** | **Previous genus** |  |
| --- | --- | --- | --- |
| Family | Chromodorididae | Bergh, 1891 |  |
| **Genus** | ***Cadlinella*** | **Thiele, 1931** |  |
|  | ***Cadlinella ornatissima*** | **(Risbec, 1928)** |  |
|  | *Cadlinella subornatissima* | Baba, 1996 |  |
| Hypothesized members | | | |
|  | *Cadlinella hirsuta* | Rudman, 1995 |  |
| **Genus** | ***Tyrinna*** | **Bergh, 1898** |  |
|  | *Tyrinna evelinae* | (Marcus, 1958) |  |
|  | ***Tyrinna nobils*** | **Bergh, 1898** |  |
| **Genus** | ***Noumea*** | **Risbec, 1928** |  |
|  | *Noumea alboannulata* | Rudman, 1986 |  |
|  | *Noumea haliclona* | (Burn, 1957) |  |
|  | *Noumea laboutei* | Rudman, 1986 |  |
|  | *Noumea norba* | Marcus & Marcus, 1970 |  |
|  | *Nomea protea* | Gosliner, 1994 |  |
|  | *Noumea purpurea* | Baba, 1949 |  |
|  | ***Noumea romeri*** | **Risbec, 1928** |  |
|  | *Noumea simplex* | (Pease, 1871) |  |
|  | *Noumea varians* | (Pease, 1871) |  |
|  | *Noumea verconis* | (Basedow & Hedley, 1905) | *Verconia* |
| Hypothesized members | | | |
|  | *Noumea decussata* | Risbec, 1928 |  |
|  | *Noumea hongkongensis* | Rudman, 1990 |  |
|  | *Noumea nivalis* | Baba, 1937 |  |
|  | *Noumea subnivalis* | Baba, 1987 |  |
|  | *Noumea sudanica* | Rudman, 1985 |  |
|  | *Noumea vericoniforma* | Rudman, 1995 |  |
|  | **Name** | **Authority** | **Previous genus** |
| **Genus** | ***Diversidoris*** | **Rudman, 1987** |  |
|  | ***Diversidoris aurantionodulosa*** | **Rudman, 1987** |  |
|  | *Diversidoris crocea* | (Rudman, 1986) | *Noumea* |
|  | *Diversidoris flava* | (Rudman, 1986) | *Noumea* |
|  | *Diversidoris* sp.2937 | Moorea BioCode |  |
| Hypothesized members | | |  |
|  | *Diversidoris sulphera* | (Rudman, 1986) | *Noumea* |
| **Genus** | **Glossodoris** | **Ehrenberg, 1831** |  |
|  | *Glossodoris cincta* | (Bergh, 1889) |  |
|  | *Glossodoris* cf. *cincta* | *Madagascar* |  |
|  | *Glosssodoris hikuerensis* | (Pruvot-Fol, 1954) |  |
|  | **Name** | **Authority** | **Previous genus** |
|  | ***Glossodoris pallida*** | **Ruppell & Leuckart, 1828** |  |
|  | *Glossodoris rufomarginata* | (Bergh, 1890) |  |
| Hypothesized members | | | |
|  | *Glossodoris angasi* | Rudman, 1986 |  |
|  | *Glossodoris gregoria* | Rudman, 1986 |  |
|  | *Glossodoris vespa* | (Rudman, 1990) |  |
| **Genus** | ***Ardeadoris*** | **Rudman, 1984** |  |
|  | *Ardeadoris angulstolutea* | (Rudman, 1990) | *Noumea* |
|  | *Ardeadoris averni* | (Rudman, 1985) | *Glossodoris* |
|  | ***Ardeadoris egretta*** | **Rudman, 1984** |  |
|  | *Aredadoris pullata* | (Rudman, 1995) | *Glossodoris* |
|  | *Ardeadoris rubroannulata* | (Rudman, 1986) | *Glossodoris* |
|  | *Ardeadoris scottjohnsoni* | Bertsch & Gosliner, 1989 |  |
|  | *Ardeadoris tomsmithi* | (Bertsch & Gosliner, 1989) | *Glossodoris* |
|  | *Ardeadoris undaura* | (Rudman, 1985) | *Glossodoris* |
| Hypothesized members | | | |
|  | **Name** | **Authority** | **Previous genus** |
|  | *Ardeadoris carlsoni* | (Rudman, 1986) | *Glossodoris* |
|  | *Ardeadoris cruenta* | (Rudman, 1986) | *Glossodoris* |
|  | *Ardeadoris electra* | Rudman, 1990 | *Glossodoris* |
|  | *Ardeadoris poliahu* | (Bertsch & Gosliner, 1989) | *Glossodoris* |
|  | *Ardeadoris symmetrica* | Rudman, 1990 | *Glossodoris* |
| **Genus** | ***Chromodoris*** | **Alder & Hanncock, 1855** |  |
|  | *Chromodoris africana* | Eliot, 1904 |  |
|  | *Chromodoris annae* | (Bergh, 1877) |  |
|  | *Chromodoris aspersa* | (Gould, 1852) |  |
|  | *Chromodoris boucheti* | Rudman, 1982 |  |
|  | *Chromodoris burni* | Rudman, 1982 |  |
|  | *Chromodoris colemani* | Rudman, 1982 |  |
|  | *Chromodoris dianae* | Gosliner & Behrens, 1998 |  |
|  | *Chromodoris elisabethina* | Bergh, 1877 |  |
|  | *Chromodoris hamiltoni* | Rudman, 1977 |  |
|  | *Chromodoris joshi* | Gosliner & Behrens, 1998 |  |
|  | *Chromodoris kuiteri* | Rudman, 1982 |  |
|  | *Chromodoris lochi* | Rudman, 1982 |  |
|  | ***Chromodoris magnifica*** | **(Quoy & Gaimard, 1832)** |  |
|  | *Chromodoris michaeli* | Gosliner & Behrens, 1998 |  |
|  | *Chromodoris quadricolor* | (Ruppell & Leuckart, 1828) |  |
|  | *Chromodoris striatella* | Bergh, 1876 |  |
|  | **Name** | **Authority** | **Previous genus** |
|  | *Chromodoris strigata* | Rudman, 1982 |  |
|  | *Chromodoris westraliensis* | (O’Donoghue, 1924) |  |
|  | *Chromodoris willani* | Rudman, 1982 |  |
| Hypothesized members | | | |
|  | *Chromodoris buchananae* | Gosliner & Behrens, 2000 |  |
|  | *Chromodoris lineolata* | (van Hasslet, 1824) |  |
|  | *Chromodoris mandapamensis* | Valdés, Mollo & Ortea, 1999 |  |
|  | *Chromodoris orientalis* | Rudman, 1983 |  |
| **Genus** | ***Goniobranchus*** | **Pease, 1866** |  |
|  | *Goniobranchus cf. alderi* | Collingwoodi, 1881 | *Chromodoris* |
|  | *Goniobranchus albopunctatus* | (Pease, 1860) | *Chromodoris* |
|  | *Goniobranchus albopustulosus* | (Rudman, 1985) | *Chromodoris* |
|  | *Goniobranchus coi* | (Risbec, 1956) | *Chromodoris* |
|  | *Goniobranchus collingwoodi* | (Rudman, 1987) | *Chromodoris* |
|  | *Goniobranchus* cf. *collingwoodi* | (Rudman, 1987) | *Chromodoris* |
|  | *Goniobranchus daphne* | (Angus, 1864) | *Chromodoris* |
|  | *Goniobranchus decorus* | (Pease, 1860) | *Chromodoris* |
|  | *Goniobranchus epicurius* | (Basedow & Hedley, 1905) | *Chromodoris* |
|  | *Goniobranchus fidelis* | (Kelaart, 1858) | *Chromodoris* |
|  | *Goniobranchus geminus* | (Rudman, 1987) | *Chromodoris* |
|  | *Goniobranchus geometricus* | (Risbec, 1928) | *Chromodoris* |
|  | *Goniobranchus heatherae* | (Gosliner, 1994) | *Chromodoris* |
|  | *Goniobranchus hintuanensis* | (Gosliner & Behrens, 1998) | *Chromodoris* |
|  | *Goniobranchus kuniei* | (Pruvot-Fol, 1930) | *Chromodoris* |
|  | *Goniobranchus preciosus* | (Kelaart, 1858) | *Chromodoris* |
|  | *Goniobranchus reticulatus* | (Quoy & Gaimard, 1832) | *Chromodoris* |
|  | *Goniobranchus roboi* | (Gosliner & Behrens, 1998) | *Chromodoris* |
|  | *Goniobranchus* cf. *roboi* | (Gosliner & Behrens, 1998) | *Chromodoris* |
|  | *Goniobranchus sinensis* | (Rudman, 1985) | *Chromodoris* |
|  | *Goniobranchus verrieri* | (Crosse, 1875) | *Chromodoris* |
|  | ***Goniobranchus vibratus*** | **(Pease, 1860)** | *Chromodoris* |
| Hypothesized members | | | |
|  | *Goniobranchus albonares* | (Rudman, 1990) | *Chromodoris* |
|  | *Goniobranchus alius* | (Rudman, 1987) | *Chromodoris* |
|  | *Goniobranchus annulatus* | (Eliot, 1904) | *Chromodoris* |
|  | *Goniobranchus aureomarginatus* | Cheeseman, 1881 | *Chromodoris* |
|  | *Goniobranchus aureopurpureus* | (Collingwood, 1881) | *Chromodoris* |
|  | *Goniobranchus aurigerum* | (Rudman, 1990) | *Chromodoris* |
|  | *Goniobranchus cavae* | (Eliot, 1904) | *Chromodoris* |
|  | **Name** | **Authority** | **Previous genus** |
|  | *Goniobranchus cazae* | (Gosliner & Behrens, 1998) | *Chromodoris* |
|  | *Goniobranchus charlottae* | (Schrödel, 1999) | *Chromodoris* |
|  | *Goniobranchus conchyliatus* | (Yonow, 1984) | *Chromodoris* |
|  | *Goniobranchus hunterae* | Rudman, 1983 | *Chromodoris* |
|  | *Goniobranchus galactos* | (Rudman & Johnson, 1985) | *Chromodoris* |
|  | *Goniobranchus gleniei* | (Kelaart, 1858) | *Chromodoris* |
|  | *Goniobranchus kitae* | (Gosliner, 1994) | *Chromodoris* |
|  | *Goniobranchus lekker* | (Gosliner, 1994) | *Chromodoris* |
|  | *Goniobranchus loringi* | (Angus, 1864) | *Chromodoris* |
|  | *Goniobranchus multimaculosus* | (Rudman, 1987) | *Chromodoris* |
|  | *Goniobranchus naiki* | (Valdés, Mollo & Oreta, 1999) | *Chromodoris* |
|  | *Goniobranchus obsoletus* | (Ruppell & Leuckart, 1831) | *Chromodoris* |
|  | *Goniobranchus petechialis* | (Gould, 1852) | *Chromodoris* |
|  | *Goniobranchus pruna* | (Gosliner, 1994) | *Chromodoris* |
|  | *Goniobranchus rubrocornutus* | (Rudman, 1985) | *Chromodoris* |
|  | *Goniobranchus rufomaculatus* | (Pease, 1871) | *Chromodoris* |
|  | *Goniobranchus setoensis* | (Baba, 1938) | *Chromodoris* |
|  | *Goniobranchus tennentanus* | (Kelaart, 1859) | *Chromodoris* |
|  | *Goniobranchus tinctorius* | (Ruppell & Leuckart, 1828) | *Chromodoris* |
|  | *Goniobranchus trimarginatus* | (Winckworth, 1946) | *Chromodoris* |
|  | *Goniobranchus tritos* | (Yonow, 1994) | *Chromodoris* |
|  | *Goniobranchus tumulifera* | (Collingwood, 1881) | *Chromodoris* |
|  | *Goniobranchus woodwardae* * | (Rudman, 1983) | *Chromodoris* |
| **Genus** | ***‘Doriprismatica’*** | **d’Orbigny, 1839** |  |
|  | ***‘Dorisprismatica’ atromarginata*** | **(Cuvier, 1804)** | *Glossodoris* |
|  | *‘Dorisprismatica’ kulonba* | (Burn, 1966) | *Digidentis* |
|  | *‘Dorisprismatica’ sedna* | (Marcus & Marcus, 1967) | *Glossodoris* |
|  | *‘Dorisprismatica’ sibogae* | (Bergh, 1905) | *Glossodoris* |
|  | *‘Dorisprismatica’ stellata* | (Rudman, 1986) | *Glossodoris* |
| Hypothesized members | | | |
|  | *‘Dorisprismatica’ dendrobranchia* | (Rudman, 1990) | *Glossodoris* |
|  | *‘Dorisprismatica’ paladentata* | (Rudman, 1986) | *Glossodoris* |
|  | *‘Doriprsimatica’ plumbea* | (Pagenstecher, 1877) | *Glossodoris* |
|  | *‘Dorisprismatica’ tibboeli* | (Valdés & Adams, 2005) | *Glossodoris* |
| **Genus** | ***‘Felimida’*** | **Marcus 1971** |  |
|  | *‘Felimida’ baumanni* | (Bertsch, 1970) | *Glossodoris* |
|  | *‘Felimida’ clenchi* | (Russell, 1935) | *Chromodoris* |
|  | *‘Felimida’ dalii* | (Bergh, 1879) | *Glossodoris* |
|  | *‘Felimida’ edmundsi* | (Cervera, Garcia-Gomez & Ortea, 1989) | *Glossodoris* |
|  | **Name** | **Authority** | **Previous genus** |
|  | *‘Felimida’ krohni* | (Verany, 1846) | *Chromodoris* |
|  | *‘Felimida’ luteorosea* | (von Rapp, 1827) | *Chromodoris* |
|  | *‘Felimida’ norrisi* | (Farmer, 1963) | *Chromodoris* |
|  | *‘Felimida’ ocellata* | (Ortea, J.A., S. Gofás & Á. Valdés, 1997) | *Chromodoris* |
|  | *‘Felimida’ purpurea* | (Risso in Guérin, 1831) | *Chromodoris* |
|  | *‘Felimida’ socorroensis* | (Behrens, Gosliner & Hermisillo, 2009) | *Chromodoris* |
|  | ***‘Felimida’ sphoni*** | **Marcus, Ev., 1971** | *Chromodoris* |
| Hypothesized members | | | |
|  | *‘Felimida’ binza* | (Marcus & Marcus, 1963) | *Chromodoris* |
|  | *‘Felimida’ britoi* | (Ortea & Perez, 1983) | *Chromodoris* |
|  | *‘Felimida’ corimbae* | (Ortea, J.A., S. Gofás & Á. Valdés, 1997) | *Chromodoris* |
|  | *‘Felimida’ galexorum* | (Bertsch, 1978) | *Chromodoris* |
|  | *‘Felimida’ goslineri* | (Ortea & Valdés *in* Ortea, Valdés & Garcia-Gomez, 1996) | *Chromodoris* |
|  | *‘Felimida’ ghanensis* | (Edmunds, 1968) | *Glossodoris* |
|  | *‘Felimida’ kpone* | (Edmunds, 1981) | *Chromodoris* |
|  | *‘Felimida’ luteopunctata* | (Gantes, 1962) | *Chromodoris* |
|  | *‘Felimida’ macfarlandi* | (Cockerell, 1901) | *Chromodoris* |
|  | *‘Felimida’ marislae* | (Bertsch, 1973) | *Chromodoris* |
|  | *‘Felimida’ neona* | Marcus, 1955 | *Chromodoris* |
|  | *‘Felimida’ ponga* | (Marcus & Marcus, 1970) | *Chromodoris* |
|  | *‘Felimida’ punctilucens* | (Bergh, 1890) | *Glossodoris* |
|  | *‘Felimida’ regalis* | Ortea, Caballer & Moro, 2001 | *Chromodoris* |
|  | *‘Felimida’ rodomaculata* | (Ortea & Valdés, 1992) | *Chromodoris* |
|  | *‘Felimida’ rolani* | (Ortea, 1988) | *Chromodoris* |
|  | *‘Felimida’ ruzafai* | (Ortea, Bacallado & Valdés, 1992) | *Chromodoris* |
|  | *‘Felimida’ socorroensis* | (Behrens, Gosliner & Hermisillo, 2009) | *Chromodoris* |
| **Sub-Family** | **Miamirinae** | **Bergh 1891** |  |
| **Genus** | ***Miamira*** | **Bergh, 1875** |  |
|  | *Miamira alleni* | (Gosliner, 1996) | *Ceratosoma* |
|  | *Miamira magnifica* | (Eliot, 1910) | *Ceratosoma* |
|  | *Miamira miramirana* | (Bergh, 1875) | *Ceratosoma* |
|  | ***Miamira sinuata* =**  ***Miamira nobilis*** | **(van Hasselt, 1824)**  **Bergh, 1874** | *Ceratosoma* |
| Hypothesized members | | | |
|  | *Miamira flavicostata* | (Baba, 1940) | *Ceratosoma* |
|  | *Miamira moloch* | (Rudman, 1988) | *Ceratosoma* |
| **Genus** | ***Ceratosoma*** | **Adams and Reeve, 1850** |  |
|  | *Ceratosoma amoenum* | (Cheeseman, 1886) |  |
|  | *Ceratosoma ingozi* | Gosliner, 1996 |  |
|  | **Name** | **Authority** | **Previous genus** |
|  | *Ceratosoma gracillimum* | Semper *in* Bergh, 1876 |  |
|  | *Ceratosoma tenue* | Abraham, 1876 |  |
|  | ***Ceratosoma trilobatum* =** | **(J.E.Gray, 1827)** |  |
|  | ***Ceratosoma corigerum*** | **Adams & Reeve, 1850** |  |
|  | *Ceratosoma sp. 2* | *in* Gosliner, Behrens and Valdés, 2008 |  |
| Hypothesized members | | | |
|  | *Ceratosoma bicolor* | Baba, 1949 |  |
|  | *Ceratosoma brevicaudatum* | Abraham, 1876 |  |
|  | *Ceratosoma palliolatum* | Rudman, 1988 |  |
| **Genus** | ***Felimare*** | **Marcus & Marcus 1967** |  |
|  | *Felimare agassizii* | (Bergh, 1894) | *Hypselodoris* |
|  | ***Felimare bayeri*** | **Marcus & Marcus, 1967** | *Hypselodoris* |
|  | *F Felimare bilineata* | (Pruvot-Fol, 1953) | *Hypselodoris* |
|  | *Felimare californiensis* | (Bergh, 1879) | *Hypselodoris* |
|  | *Felimare elegans picta* | (Schultz, 1836) | *Hypselodoris* |
|  | *Felimare kempfi* | (Ev. Marcus, 1970) | *Hypselodoris* |
|  | *Felimare lapislazuli* | (Bertsch & Ferreira, 1971) | *Hypselodoris* |
|  | *Felimare midatlantica* | (Gosliner, 1990) | *Hypselodoris* |
|  | *Felimare marci* | (Ev. Marcus, 1970) | *Hypselodoris* |
|  | ***Felimare orsinii*** | **(Verany, 1846)** | *Hypselodoris* |
|  | *Felimare porterae* | (Cockerell, 1902) | *Hypselodoris* |
|  | *Felimare ruthae* | (Marcus & Hughes, 1974) | *Hypselodoris* |
|  | *Felimare villafranca* | (Risso, 1818) | *Hypselodoris* |
|  | *Felimare zebra* | (Heilprin, 1889) | *Hypselodoris* |
| Hypothesized members | | | |
|  | *Felimare acriba* | (Marcus & Marcus, 1967) | *Hypselodoris* |
|  | *Felimare amalguae* | (Gosliner & Bertsch, 1988) | *Mexichromis* |
|  | *Felimare azorica* | (Ortea, Valdés & Garcia-Gomez, 1996) | *Hypselodoris* |
|  | *Felimare cantabrica* | (Bouchet & Ortea, 1980) | *Hypselodoris* |
|  | *Felimare ciminoi* | (Ortea & Valdés in Ortea, Valdés & Garcia-Gomez, 1996) | *Hypselodoris* |
|  | *Felimare espinosai* | (Ortea & Valdés in Ortea, Valdés & Garcia-Gomez, 1996) | *Hypselodoris* |
|  | *Felimare fontandraui* | (Pruvot-Fol, 1951) | *Hypselodoris* |
|  | *Felimare francoisae* | (Bouchet in Bouchet & Ortea, 1980) | *Hypselodoris* |
|  | *Felimare gasconi* | (Ortea in Ortea, Valdés & Garcia-Gomez, 1996) | *Hypselodoris* |
|  | *Felimare ghiselini* | (Bertsch, 1978) | *Hypselodoris* |
|  | *Felimare gofasi* | (Ortea & Valdés in Ortea, Valdés & Garcia-Gomez, 1996) | *Hypselodoris* |
|  | *Felimare juliae* | (DaCosta, Padula & Schrodel, 2010) | *Hypselodoris* |
|  | *Felimare lajensis* | (Troncoso, Garcia & Urgorri, 1998) | *Hypselodoris* |
|  | *Felimare lilyeveae* | (Alejandrino & Valdés, 2006) | *Hypselodoris* |
|  | **Name** | **Authority** | **Previous genus** |
|  | *Felimare malacitana* | (Luque, 1986) | *Hypselodoris* |
|  | *Felimare molloi* | (Ortea & Valdés in Ortea, Valdés | *Hypselodoris* |
|  | *Felimare muniainae* | (Ortea & Valdés in Ortea, Valdés & Garcia-Gomez, 1996) | *Hypselodoris* |
|  | *Felimare olgae* | (Ortea & Bacallalo, 2007) | *Hypselodoris* |
|  | *Felimare picta verdensis* | (Ortea, Valdés & Garcia-Gomez, 1996) | *Hypselodoris* |
|  | *Felimare pinna* | (Ortea, 1988) | *Hypselodoris* |
|  | *Felimare sycilla* | (Bergh, 1890) | *Hypselodoris* |
|  | *Felimare tema* | (Edmunds, 1981) | *Hypselodoris* |
|  | *Felimare xicoi* | (Ortea & Valdés in Ortea, Valdés & Garcia-Gomez, 1996) | *Hypselodoris* |
| **Genus** | ***Hypselodoris*** | **Stimpson 1855** |  |
|  | *Hypselodoris apolegma* | (Yonow, 2001) |  |
|  | *Hypselodoris bennetti* | (Angus, 1864) |  |
|  | *Hypselodoris bertschi* | Gosliner & Johnson, 1999 |  |
|  | *Hypselodoris bollandi* | Gosliner & Johnson, 1999 |  |
|  | *Hypselodoris bullocki* | (Collingwoodi, 1881) |  |
|  | *Hypselodoris capensis* | (Barnard, 1927) |  |
|  | *Hypselodoris emma* | Rudman, 1977 |  |
|  | *Hypselodoris infucata* | (Ruppell & Leuckart, 1828) |  |
|  | *Hypselodoris jacksoni* | Wilson & Wilan, 2007 |  |
|  | *Hypselodoris kaname* | Baba, 1994 |  |
|  | *Hypselodoris krakatoa* | Gosliner & Johnson, 1999 |  |
|  | *Hypselodoris maculosa* | (Pease, 1871) |  |
|  | *Hypselodoris*  cf. *maculosa* | (Pease, 1871) |  |
|  | *Hypselodoris maridadilus* | Rudman, 1977 |  |
|  | *Hypselodoris maritima* | (Baba, 1949) |  |
|  | *Hypselodoris*  cf. *nigrolineata* | (Eliot, 1904) |  |
|  | ***Hypselodoris obscura*** | **Stimpson, 1855** |  |
|  | *Hypselodoris paulinae* | Gosliner & Johnson, 1999 |  |
|  | *Hypselodoris peasei* | (Bergh, 1860) |  |
|  | *Hypselodoris purpureomaculosa* | (Hamatani, 1995) |  |
|  | *Hypselodoris reidi* | Gosliner & Johnson, 1999 |  |
|  | *Hypselodoris whitei* | (Adams & Reeve, 1850) |  |
|  | *Hypselodoris zephyra* | Gosliner & Johnson, 1999 |  |
|  | **Risbecia clade** | | |
|  | *Hypselodoris imperialis* | (Pease, 1860) | *Risbecia* |
|  | *Hypselodoris pulchella* | (Ruppell & Leuckart, 1828) | *Risbecia* |
|  | *Hypselodoris tryoni* | (Garrett, 1873) | *Risbecia* |
| Hypothesized members | | | |
|  | *Hypselodoris alboterminata* | Gosliner & Johnson, 1999 |  |
|  | **Name** | **Authority** | **Previous genus** |
|  | *Hypselodoris babai* | Gosliner & Behrens, 2000 |  |
|  | *Hypselodoris carnea* | (Bergh, 1889) |  |
|  | *Hypselodoris dollfusi* | (Pruvot-Fol, 1933) |  |
|  | *Hypselodoris festiva* | (A.Angus, 1861) |  |
|  | *Hypselodoris flavomarginata* | Rudman, 1995 |  |
|  | *Hypselodoris fucata* | Gosliner & Johnson, 1999 |  |
|  | *Hypselodoris iacula* | Gosliner & Johnson, 1999 |  |
|  | *Hypselodoris insulana* | Gosliner & Johnson, 1999 |  |
|  | *Hypselodoris kanga* | Rudman, 1977 |  |
|  | *Hypselodoris lacteola* | Rudman, 1995 |  |
|  | *Hypselodoris nigrolineata* | (Eliot, 1904) |  |
|  | *Hypselodoris nigrostriata* | Rudman, 1977 |  |
|  | *Hypselodoris placida* | (Baba, 1949) |  |
|  | *Hypselodoris regina* | Marcus & Marcus, 1970 |  |
|  | *Hypselodoris rudmani* | Gosliner & Johnson, 1999 |  |
|  | *Hypselodoris sagamiensis* | (Baba, 1949) |  |
|  | *Hypselodoris saintvincentius* | (Burn, 1962) |  |
|  | *Hypselodoris violabranchia* | Gosliner & Johnson, 1999 |  |
|  | *Hypselodoris zebrina* | (Alder & Hancock, 1864) |  |
|  | ***Risbecia* clade** | | |
|  | *Hypselodoris ghardaqana* | (Gohar & Aboul-Ela, 1957) | *Risbecia* |
| **Genus** | ***Mexichromis*** | **Bertsch, 1977** |  |
|  | ***Mexichromis antonii*** | **(Bertsch, 1976)** |  |
|  | *Mexichromis aurora* | (Johnson & Gosliner, 1998) | *Pectenodoris* |
|  | *Mexichromis lemniscata* | (Quoy & Gaimard, 1832) | *Durvilledoris* |
|  | *Mexichromis mariei* | (Crosse, 1872) |  |
|  | *Mexichromis macropa* | (Rudman, 1983) |  |
|  | *Mexichromis multituberculata* | (Baba, 1953) |  |
|  | *Mexichromis pusilla* | (Bergh, 1874) | *Durvilledoris* |
|  | *Mexichromis similaris* | (Rudman, 1986) | *Durvilledoris* |
|  | *Mexichromis trilineata* | (Adams & Reeve, 1850) | *Pectenodoris* |
| Hypothesized members | | | |
|  | *Mexichromis festiva* | (Angus, 1864) |  |
|  | *Mexichromis tica* | Gosliner, Ortea & Valdés, 2004 |  |
|  | *Mexichromis tura* | (Marcus & Marcus, 1967) |  |
| **Genus** | ***Thorunna*** | **Bergh, 1878** |  |
|  | *Thorunna arbuta* | (Burn, 1961) | *Digidentis* |
|  | *Thorunna australis* | (Risbec, 1928) |  |
|  | *Thorunna* cf*. australis* | (Risbec, 1928) |  |
|  | **Name** | **Authority** | **Previous genus** |
|  | *Thorunna daniellae* | (Kay & Young, 1969) |  |
|  | *Thorunna florens* | (Baba, 1949) |  |
|  | ***Thorunna furtiva*** | **Bergh, 1878** |  |
|  | *Thorunna halourga* | Johnson & Gosliner, 2001 |  |
|  | *Thorunna montrouzueri* | Rudman, 1996 |  |
|  | *Thorunna perplexa* | (Burn, 1957) | *Digidentis* |
|  | *Thorunna punicea* | (Rudman, 1995) |  |
|  | *Thorunna purpureopedis* | Rudman & Johnson, 1985 |  |
| Hypothesized members | | | |
|  | *Thorunna africana* | Rudman, 1984 |  |
|  | *Thorunna horologica* | Rudman, 1984 |  |
|  | *Thorunna kahuna* | Johnson & Gosliner, 2001 |  |
|  | *Thorunna speciosa* | Rudman, 1990 |  |
|  | ***Incertae sedis*** | |  |
|  | Chromodorididae *alternata* | (Burn, 1957) | *Chromodoris* |
|  | Chromodorididae *ambiguus* | (Rudman, 1987) | *Chromodoris* |
| Hypothesized | | | |
|  | Chromodorididae *albofimbria* | (Rudman, 1995) | *Durvilledoris* |
|  | Chromodorididae *circumflavus* | (Rudman, 1990) | *Durvilledoris* |
|  | Chromodorididae *aurolea* | (Rudman, 1995) | *Glossodoris* |
|  | Chromodorididae *aeruginosa* | (Rudman, 1995) | *Glossodoris* |
|  | Chromodorididae *misakinosibogae* | (Baba, 1988) | *Glossodoris* |
|  | Chromodorididae *nyalya* | (Marcus & Marcus, 1967) | *Risbecia* |
|  | Chromodorididae *catalai* | (Rudman, 1990) | *Noumea* |
|  | Chromodorididae *closei* | (Rudman, 1986) | *Noumea* |

* Possibly in a clade with Chromodorididae *ambiguus* (Rudman, 1987) and Chromordorididae *alternata* (Burn, 1957)
